# The short-term economic consequences of COVID-19: Exposure to disease, remote work and government response

**DOI:** 10.1371/journal.pone.0270341

**Published:** 2023-03-15

**Authors:** Louis-Philippe Beland, Abel Brodeur, Taylor Wright

**Affiliations:** 1 Department of Economics, Carleton University, Ottawa, Ontario, Canada; 2 Department of Economics, University of Ottawa, Ottawa, Ontario, Canada; 3 Department of Economics, Brock University, St. Catharines, Ontario, Canada; FAME|GRAPE, POLAND

## Abstract

We examine the determinants of the consequences of COVID-19 on employment and wages in the United States. Guided by a pre-analysis plan, we investigate whether the economic consequences of COVID-19 were larger for certain occupations, using four indexes: workers relatively more exposed to disease, workers that work with proximity to coworkers, essential/critical workers and workers who can easily work remotely. We find that individuals that work in proximity to others are more affected while individuals able to work remotely and essential workers are less affected by the pandemic. We also present suggestive evidence that our indexes are likely explanations why certain demographic groups such as younger and minority workers have worse labor market outcomes during the pandemic.

## Introduction

The COVID-19 pandemic, though far from over, has already had vast human consequences. As of mid-October 2020, there were over 40,000,000 confirmed cases and about 1,100,000 fatalities worldwide. In addition to being a human tragedy, COVID-19 is also an economic one. Evidence of the catastrophic impacts of COVID-19 and non-pharmaceutical interventions such as lockdowns is by now voluminous, with many modelling scenarios predicting a long recession. However, these economic impacts are not homogeneous: as of September, 2020 the national unemployment rate was 8.4 percent, with striking differences across occupations, industries and states. This paper aims to further identify who COVID-19 and lockdowns hit hardest economically and why.

Guided by a pre-analysis plan, we investigate the following major research questions: 1) What are the short-term impacts of COVID-19 on employment and wages? 2) Are the short-term impacts of COVID-19 larger for relatively more “risky” occupations? 3) Are these effects of COVID-19 smaller for individuals who can more easily work from home? 4) Are essential workers differently impacted by COVID-19? 5) What occupations see the largest changes in economic outcomes following lockdowns? We document and explore several channels determining the short-term economic consequences of COVID-19 on labor market outcomes in the United States. Understanding these channels allows for effective policy responses. We adapt and analyze four indexes based on general occupational characteristics and tasks that are shared across occupations to explain why some workers in specific occupations, demographics, and socioeconomic strata are hit harder than others by COVID-19. Specifically, our indexes measure: workers’ ability to work remotely (adapted from [1]), workers’ exposure to infection and disease, workers’ physical proximity to coworkers, and workers’ essential/critical worker designation (See the Data and Identification Strategy section for more details). Each of these indexes captures occupational tasks and characteristics that have taken on large roles during the pandemic; companies rush to transition to working from home, shortages of personal protective equipment emerge, governments release physical distancing guidelines, and essential workers gain exemptions from lockdowns. We were the first to rely on O*NET data to create the following indexes to investigate the impacts of COVID-19: exposure to disease and proximity to coworkers. Our codes and indexes have been made publicly available since March 2020 and have been used in many other studies.

While using a pre-analysis plan is common practice now for lab and field experiments, it is less so in non-experimental settings. However, it has recently been shown that quasi-experimental studies suffer the most from p-hacking (e.g., [2]). As a rare approach to transparency in economics, we exploit the fact that the March 2020 CPS data was released only mid-April 2020, making it possible to pre-specified and publicly archived in a pre-analysis plan (PAP) our analyses prior to obtaining the data. While it was expected that COVID-19 had drastic effect on the labor market, our analysis was pre-specified before mass stay-at-home order and before potential channels of job loss were discussed. We provide more details background on our PAP in the Pre-Analysis Plan section.

We make three main contributions to the literature. First, we contribute to the estimation of the impacts of COVID-19 and lockdowns, exploring occupational characteristics as determinants of the economic consequences of the pandemic. That is, our paper provides insights into why workers in specific occupations were more or less affected by COVID-19 and lockdowns, using four different indexes (workers able to work remotely, workers relatively more exposed to disease, workers that work with proximity to coworkers and essential/critical workers). Using the Current Population Survey (CPS), we provide evidence that occupations that work in proximity to others have more adverse labor market outcomes during the pandemic while occupations able to work remotely and essential workers are less affected. Our paper was the first to explore these detailed occupational channels and how they facilitate COVID-19’s impact on the labor market ([3]).

Second, we highlight a key occupational task factor explaining larger labor market effects: being in an occupation working in proximity to others. We also document the effect of the pandemic on labor market outcomes of critical workers in the health sector versus non health sector and find lower unemployment decrease in the health care sector. Overall, we find that the labor market effects of the pandemic are larger in areas with larger number of cases and deaths.

Finally, we use our indexes to investigate why certain demographic groups are more affected after the COVID-19 outbreak. We present suggestive evidence that the work from home capacity and working in close proximity to coworkers are likely explanations why certain demographic groups such as younger and minority workers have worse labor market outcomes during the pandemic.

We contribute to a rapidly growing literature specific to the COVID-19 pandemic (see [4] for a literature review). Some of the literature estimates the heterogeneous effects of COVID-19 on different subgroups (see [5–9]) finding disproportionately negative impacts especially for black and hispanic workers, younger workers, women (especially mothers) and less educated workers. Beyond the heterogeneous effects of the pandemic there is also a developing strand of work related to the evolution of the pandemic. [10] outlines a standard epidemiological model while [11] expand on it to include testing and quarantines. We also add to work evaluating economic impacts of lockdowns and other government interventions (e.g., [12–14]) by documenting the heterogeneous impacts of lockdowns across occupational characteristics and tasks. [15] use GPS data to examine the effects of stay-at-home orders on mobility and disease prevalence, finding that orders reduce mobility. [16] use unemployment insurance claims to examine the unemployment effects of stay-at-home orders and their results suggest that such orders contributed to the rise in unemployment but that unemployment still would have risen in their absence. Researchers are also exploring the macroeconomic consequences of the pandemic ([17, 18]). For example, [19] uses survey data to examine the relationship between concerns about COVID-19 and macroeconomic expectations, finding that greater concerns is tied to higher expectations about inflation and lower expectations about unemployment.

The rest of the paper is organized as follows. **Pre-Analysis Plans** outlines our pre-analysis plan and its benefits and limitations. In **Conceptual Framework**, we provide background on the plausible channels through which COVID-19 and stay-at-home orders could affect labor market outcomes. **Data and Identificaiton Strategy** details the data collection and the identification strategy. We discuss the results in **Results**. We conclude in **Conclusion**.

## Pre-analysis plans

Recent work outlines concerns with the credibility of published research in economics (and indeed the other social sciences) resulting from publication bias and p-hacking (e.g., [20, 21]). To combat these issues, we test the hypotheses and rely on the specifications detailed in our pre-analysis plans (PAP). For this project, we pre-registered our analysis prior to the release of the CPS data detailing employment statistics after the emergence of the COVID-19 pandemic. Our first pre-analysis plan was archived on March 30, 2020, at https://osf.io/c28t5/. We then released an additional PAP in May 2020 and a final PAP in June 2020. Our paper does not present results for all questions and hypotheses from our PAPs and we explain why presently. Two of the research questions and all six of the secondary hypotheses in our first PAP related to the heterogeneous effects of COVID-19. Those analyses are no longer present in this paper as they have been repeatedly addressed by other authors using similar methods and datasets (see for example [6, 8, 22]). Moreover, we have since split the original paper ([3]) into two distinct studies–one containing only the evaluation of the lockdowns and the current paper containing the analysis pre-specified in March 2020 along with the associated lockdown related extensions from May 2020.

It is important to note that our PAPs do not specify the primary equation underlying the estimates presented in our main tables. Instead, the PAPs make clear that we intend to answer specific questions, pre-specifies the baseline regression equations that we then alter and use to answer our questions relating to our indexes. We thus highlight throughout the paper the analyses that were pre-specified and those that were not.

## Conceptual framework

Cancellations of trade shows, conventions and festivals, schools, daycare centers and other educational institutions will likely have a large negative impact on economic activity, especially for firms that require close physical proximity to other workers or clients ([23]). There is now growing evidence that a significant proportion of cases are related to occupational exposure, suggesting that certain occupations are now becoming riskier than others ([24]). In other words, occupational characteristics, such as interacting with the public and being in contact with other workers, may thus be correlated to the likelihood of contracting the disease. We test throughout whether the economic impacts of the pandemic and stay-at-home orders are related to how ‘risky’ an occupation is. On the one hand, there may be a wage premium for workers in these occupations due to the sudden increase in risk (e.g., [25]). On the other hand, some workers might decide to stop working (or forced to) given the increasing risk ([26]). These two forces could lead to a decrease in the likelihood to work, but an increase in wages for workers who still work.

COVID-19 could also have an effect on the economy through mandated closure of “non-essential” industries. While the list of essential employees varies across locations, the list of essential workers typically include the following: medical and healthcare, telecommunications, information technology systems, defense, food and agriculture, transportation and logistics, energy, water and wastewater, law enforcement, and public works industries. Essential workers, and especially those in risky occupations, could be those who are compensated for the increase in risk. The pandemic could also lead to an increase in demand for health care workers to help face the crisis.

Another dimension that we test is whether occupations with relatively more workers working remotely pre-COVID-19 were less impacted. The COVID-19 outbreak and government interventions are forcing an increasingly large number of workers to work from home. In states without regulations, many companies are encouraging or mandating that staff adopt a work-from-home policy. While these government and company policies are easily applicable in many industries, it is less the case for others. For instance, the infrastructure and policy needed for remote working for high tech firms were already in place, making the adoption of such policies feasible.

Last, COVID-19 may have been beneficial to some industries, such as consumer packaged goods and heath care, because of an increase in demand. Recent reports suggest that grocery stores, drug stores and delivery companies are seeking to fill hundreds of thousands of positions because of the panic and stay-home orders. For instance, Amazon has pledged to open 100,000 new full-time and part-time positions to meet the surge in demand and to increase pay by $2/hour ([27]).

## Data and identification strategy

### COVID-19

We rely on COVID-19 data from The Atlantic’s COVID Tracking Project (https://covidtracking.com/). The database is the product of important data collection efforts relying on information from state public health authorities, or, occasionally trusted media articles and news conferences. Our sample corresponds to the last day of the week for which employment information is collected by the CPS (February–June 2020).

Table 1 presents summary statistics for our COVID-19 data. The average cumulative number of confirmed cases per 10,000 across states is 20.48 (std. dev. 34.074), while the average cumulative number of deaths per 10,000 inhabitants across states is 1.004 (std. dev. 2.163). As the crisis has evolved, the cumulative number of cases has grown and the maximum number of cases we see in a state is 377,316 while the rate per 10,000 is 194. For deaths, the maximum we see is 24,212 with a rate per 10,000 of 13.6. These states with largest numbers of cases include New York, New Jersey, and California while states with large rates per 10,000 inhabitants (over 100) include New York, New Jersey, and other North Eastern states such as Massachusetts, Rhode Island, Connecticut, and Delaware.

**Table 1:** Descriptive statistics.

	Mean	S. D.	Max	Min	Count
*Indices*					
Exposure to infection/disease index	-0.001	1.003	3.2	-0.9	3179089
Physical proximity to coworkers index	0.008	1.003	2.2	-3.5	3179089
Remote work index	-0.005	0.998	1.3	-0.8	3061498
Critical worker index	0.003	1.000	0.7	-1.5	3060155
*Labor outcomes*					
Unemployed	0.047	0.212	1.0	0.0	3158372
In labor force	0.704	0.456	1.0	0.0	4506713
Real hourly wages in constant 2018 dollars	17.776	8.846	61.4	4.8	400508
Hours worked last week	39.042	11.628	80.0	5.0	2865884
*COVID-19 outcomes*					
Cum. Positive COVID-19 Cases	14758.749	39782.060	377316.0	0.0	255
Cum. Positive COVID-19 Cases per 10,000	20.480	34.074	194.0	0.0	255
Cum. Positive COVID-19 Deaths	780.463	2481.705	24212.0	0.0	255
Cum. Positive COVID-19 Deaths per 10,000	1.004	2.163	13.6	0.0	255

Notes: Authors’ calculations. Labor force participation: individuals in the labor force were at work; held a job but were temporarily absent from work due to factors like vacation or illness; were seeking work; or were temporarily laid off from a job during the reference period. Hours work: civilians aged 16–70 who are employed and either at work or absent from work during the survey week, all jobs. Trimmed to exclude values below 1st percentile and above 99th percentile. Hourly wages: civilians aged 16–70 currently employed as wage/salary workers, paid hourly, and were in outgoing rotation groups. Excludes self-employed persons. Trimmed to exclude values below 1st percentile and above 99th percentile. Reported in 2018 constant dollars. The descriptive statistics for the labor variables of interest are from January 2016 to June 2020. Cumulative COVID-19 cases, cases per 10,000 people, deaths, and deaths per 10,000 people are the cumulative totals corresponding to the last day of the week for which employment information is collected by the CPS. For COVID-19 outcomes we average over February–June and each observation is a state-month.

### Current population survey

We match our COVID-19 data with the Current Population Survey (CPS) from Integrated Public Use Micro Samples (IPUMS). The CPS is conducted by the Bureau of Labor Statistics (BLS) and is a monthly survey of 60,000 eligible households. The CPS provides a large sample size of workers and individual characteristics such as age, education, race, and marital status and labor market characteristics such as labor force participation, employment status, hours of work, occupation and industry. The survey questions refer to activities during the week that includes the 12th of the month.

The CPS typically includes both in-person and telephone interviews. In our pre-COVID-19 sample, about 51% were collected over the phone. Unfortunately, COVID-19 had an impact on data collection. For March and April 2020, only telephone interviews were conducted and two call centers were closed. The response rate (73% in March and 70% in April) was therefore about 10–13 percentage points lower than in preceding months ([28]). Of note the response rate for households entering the sample was particularly low. Nonetheless, the BLS “was still able to obtain estimates that met [their] standards for accuracy and reliability” ([28]). In the empirical analysis, we control for whether the interview was done in-person or telephone.

Table 1 provides descriptive statistics for our labor market outcome variables of interest from January 2016 to June 2020. Our sample consists of civilians aged 16–70 over the time period. We have 3,070,317 observations for unemployment. Our sample size is smaller for hourly wages since this information is only asked of the outgoing rotation groups. Approximately 4.4% of respondents were unemployed and 71% were in the labor force. We restrict the sample to individuals working for hours of work and wages. On average, the real hourly wage (2018 dollars) was about $18 and workers were usually working 39 hours per week at all jobs.

### Occupational measures

Our occupational measures of exposure to disease or infection and physical proximity come from the Occupational Information Network (O*NET) survey data. O*NET is a program sponsored by the U.S. Department of Labor which aims to gather occupational data and develop applications to help create and maintain a skilled labor force. The survey data is collected after pre-testing survey construction and features done in conjunction with the Department of Labor. The survey uses a two-stage design. First, businesses expected to have the occupations required are randomly sampled and then workers from those business are randomly sampled and provided questionnaires.

Our measure of exposure to disease is taken from a survey question asking “How often does this job require exposure to disease/infections?” with five possible answers: (1) Never, (2) Once a year or more but not every month, (3) Once a month or more but not every week, (4) Once a week or more but not every day, and (5) Every day. The translation of these responses into an index is done by O*NET and shown in Figure S1 in S1 Appendix. The exact formula used for converting the survey responses into the index values is described in the S1 Appendix. The top and bottom 15 occupations are shown in Table 2, while Table S1 in S1 Appendix shows the middle of the distribution (i.e., 60^th^ to 40^th^ percentiles). The following four occupation codes have a score of 100: Acute care nurses, dental hygienists, family and general practitioners, and internists.

**Table 2:** Index for exposure to disease.

Occupation	Score	Occupation	Score
*Top 15*		*Bottom 15*	
Acute Care Nurses	100	Actuaries	0
Dental Hygienists	100	Aerospace Engineers	0
Family & Gen. Practitioners	100	Agents of Artists & Athletes	0
Internists, General	100	Art Directors	0
Critical Care Nurses	99	Assessors	0
Hospitalists	99	Auditors	0
Oral Surgeons	99	Automotive Engineers	0
Respiratory Therapists	98	Bicycle Repairers	0
Respiratory Therapy Technicians	98	Cabinetmakers Carpenters	0
Anesthesiologist Assistants	97	Camera & Photo Repairers	0
Occupational Therapy Aides	97	Cartographers and Photogrammetrists	0
Orderlies	97	City & Regional Planning Aides	0
Dental Assistants	96	Climate Change Analysts	0
Medical & Clinical Technologists	96	Commercial & Industrial Designers	0
Nurse Anesthetists	96	Computer Research Scientists	0

Notes: Our measure of exposure to disease is taken from a survey question asking “How often does this job require exposure to disease/infections?” with five possible answers: (1) Never, (2) Once a year or more but not every month, (3) Once a month or more but not every week, (4) Once a week or more but not every day, and (5) Every day. The translation of these responses into an index is done by O*NET.

Our measure of physical proximity is taken from a survey question asking “How physically close to other people are you when you perform your current job?” with five possible responses: (1) I don’t work near other people (beyond 100 ft.), (2) I work with others but not closely(e.g., private office), (3) Slightly close (e.g., shared office), (4) Moderately close (at arm’s length), and (5) Very close (near touching). The analogous graphic for this question is shown in Figure S2 in S1 Appendix. The top and bottom 15 occupations are shown in Table 3. The following four occupation codes have a score of 100: Choreographers, dental hygienists, physical therapists, and sports medicine physicians. Table S2 in S1 Appendix presents examples of occupations that score between the 60^th^ to 40^th^ percentiles. We convert the O*NET occupation codes into Standard Occupational Classification (SOC) codes using the crosswalks provided by O*NET.

**Table 3:** Index for physical proximity.

Occupation	Score	Occupation	Score
*Top 15*		*Bottom 15*	
Choreographers	100	Fallers	7
Dental Hygienists	100	Fine Artists (e.g., Painters)	9
Physical Therapists	100	Poets and Creative Writers	14
Sports Medicine	100	Logging Equipment Operators	14
Dental Assistants	99	Hunters and Trappers	17
Dentists, General	99	Wellhead Pumpers	19
Oral Surgeons	99	Cooks, Private Household	21
Skincare Specialists	99	Farmworkers and Laborers	24
Surgical Technologists	99	Dredge Operators	27
Urologists	99	Bridge and Lock Tenders	28
Dancers	99	Pesticide Handlers & Applicators	29
Dermatologists	98	Environmental Economists	29
Prosthodontists	98	Petroleum Engineers	30
Radiation Therapists	98	Refuse & Recyclable Collectors	31
Respiratory Therapy	98	Political Scientists	31

Notes: This index is taken from a survey question asking “How physically close to other people are you when you perform your current job?” with five possible responses: (1) I don’t work near other people(beyond 100 ft.), (2) I work with others but not closely(e.g., private office), (3) Slightly close (e.g., shared office), (4) Moderately close (at arm’s length), and (5) Very close (near touching).

We complement these indexes by using the classifications of the feasibility of working from home created by [1] and essential worker designations based on the LMI Institute index (see this link for more details: https://www.lmiontheweb.org/more-than-half-of-u-s-workers-in-essential-occupations-in-the-fight-against-covid-19/). Our PAP indicated that we would measure remote work by creating an index using the American Community Survey’s question about travel to work. We instead use the classification developed by [1]. We make this alteration to address concerns that our measure of remote work prior to COVID-19 may be capturing preferences, or wealth effects rather than the potential for occupations to be done remotely. In contrast, the [1] measure classifies occupations based on tasks and so better reflects the ease with which occupations can be done from home. Additionally, at the time of our pre-analysis plan, this classification was not available. [1] classify the feasibility of working at home in the U.S. and argue that 34% of jobs can plausibly be performed at home. [29] conducted a survey early April and found that 34% of individuals employed four weeks earlier reported they were commuting and are now working from home. The essential workers index provides a list of essential occupations in several fields: medical and healthcare, telecommunications, information technology systems, defense, food and agriculture, transportation and logistics, energy, water and wastewater, law enforcement, and public works industries.

We then merge these indexes with our data from the CPS after converting its occupation codes into SOC equivalents. In cases where the SOC codes from the CPS are at a higher level of aggregation than those of our indexes, we assign an index value based on the weighted average of the sub-occupations, weighting by each sub-occupation’s share of employment in the aggregated occupation (taken from the BLS’ Occupational Employment Statistics estimates). Table 1 provides descriptive statistics. Indexes are standardized to a mean of 0 and standard deviation of 1, to facilitate interpretation (numbers will not be exactly 0 or 1 due to rounding). Our exposure and proximity indexes take on a much wider range of values, in part because the classifications that our remote and essential worker indexes are built from are binary (except where occupations are at a higher level of aggregation).

**Fig 1:**
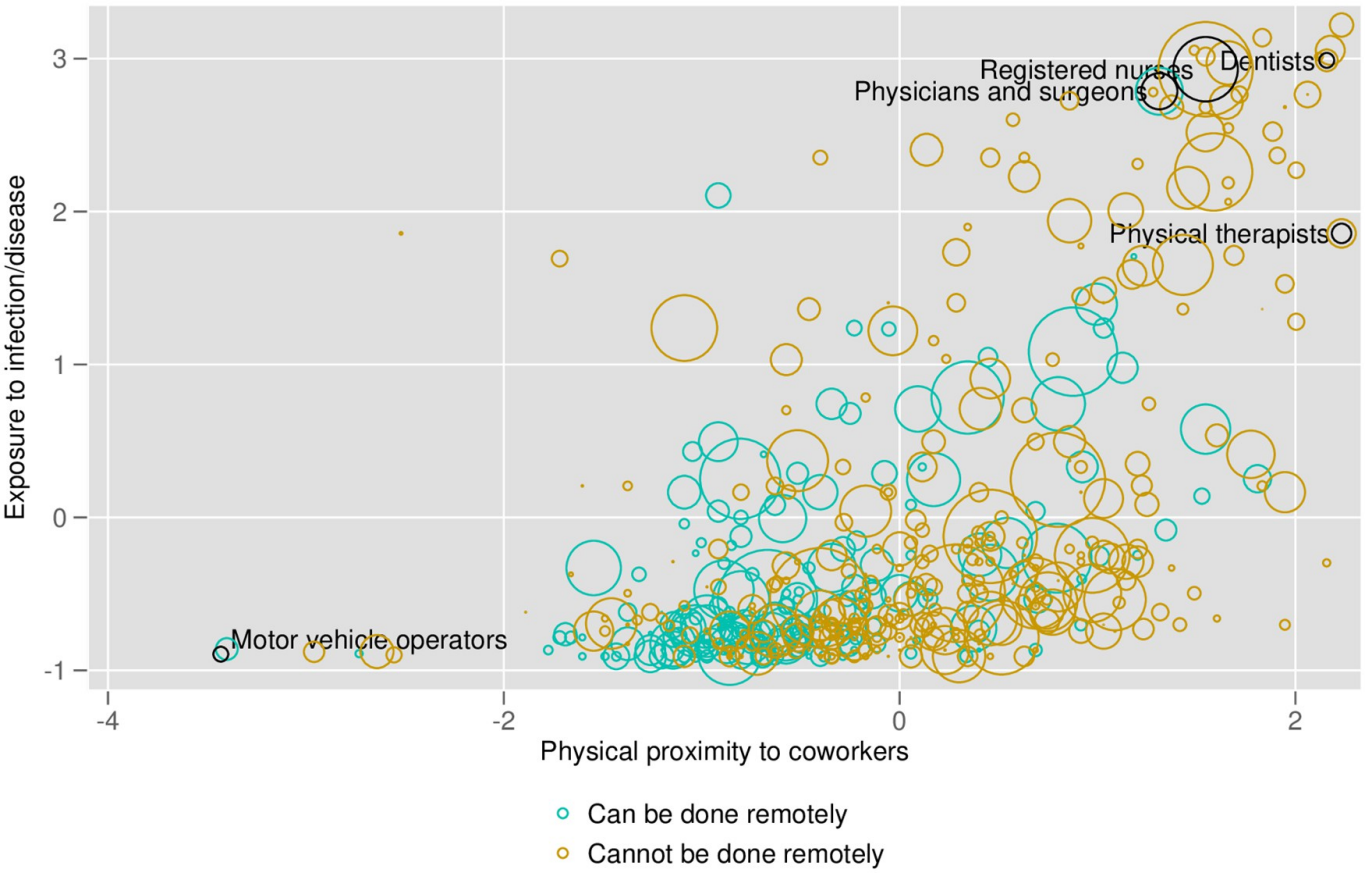
Physical proximity, exposure to the disease and remote work by occupation. Notes: Each circle represents an occupation. The size of each circle represents the number of CPS respondents employed in that occupation–the larger the circle, the greater the number of people employed in that occupation. The x-axis plots each occupation’s physical proximity to coworkers, measured by O*NET’s index. The further to the right, the closer in proximity employees in that occupation work with their coworkers. The y-axis plots each occupation’s exposure to infection and disease, also measured by O*NET’s index. The further up, the more frequently employees in that occupation are exposes to infection and disease. The color of the circles corresponds to the whether or not the occupation can be performed remotely via [1].

Fig 1 illustrates three of our indexes. Each circle in the figure represents an occupation. The size of each circle represents the number of CPS respondents employed in that occupation–the larger the circle, the greater the number of people employed in that occupation. The x-axis plots each occupation’s physical proximity to coworkers, measured by O*NET’s index. The further to the right, the closer in proximity employees in that occupation work with their coworkers. The y-axis plots each occupation’s exposure to infection and disease, also measured by O*NET’s index. The further up, the more frequently employees in that occupation are exposed to infection and disease. The color of the circles corresponds to whether or not an occupation can be done remotely. For simplicity, in this figure we code any occupation as “can be done remotely” if the share of jobs that can be done in that occupation is greater than zero. We also present variants of this in Figures S3 and S4 in S1 Appendix.

We can see a clear positive (convex) relationship between our indexes of physical proximity and exposure to infection and disease, with health workers (e.g., dentists, nurses and physicians) scoring relatively high for both indexes. The correlation between exposure and proximity is 0.548. In contrast, there is a negative correlation between remote work and exposure (correlation of -0.210), suggesting that workers in occupations requiring exposure to disease/infections are less likely to be working from home. Similarly, our remote work and proximity indexes are negatively correlated (correlation of -0.450). Our essential workers index is negatively correlated with remote work (-0.202) while being positively correlated with our exposure index (0.129) and mildly positively correlated with our proximity to coworkers index (0.098).

**Fig 2:**
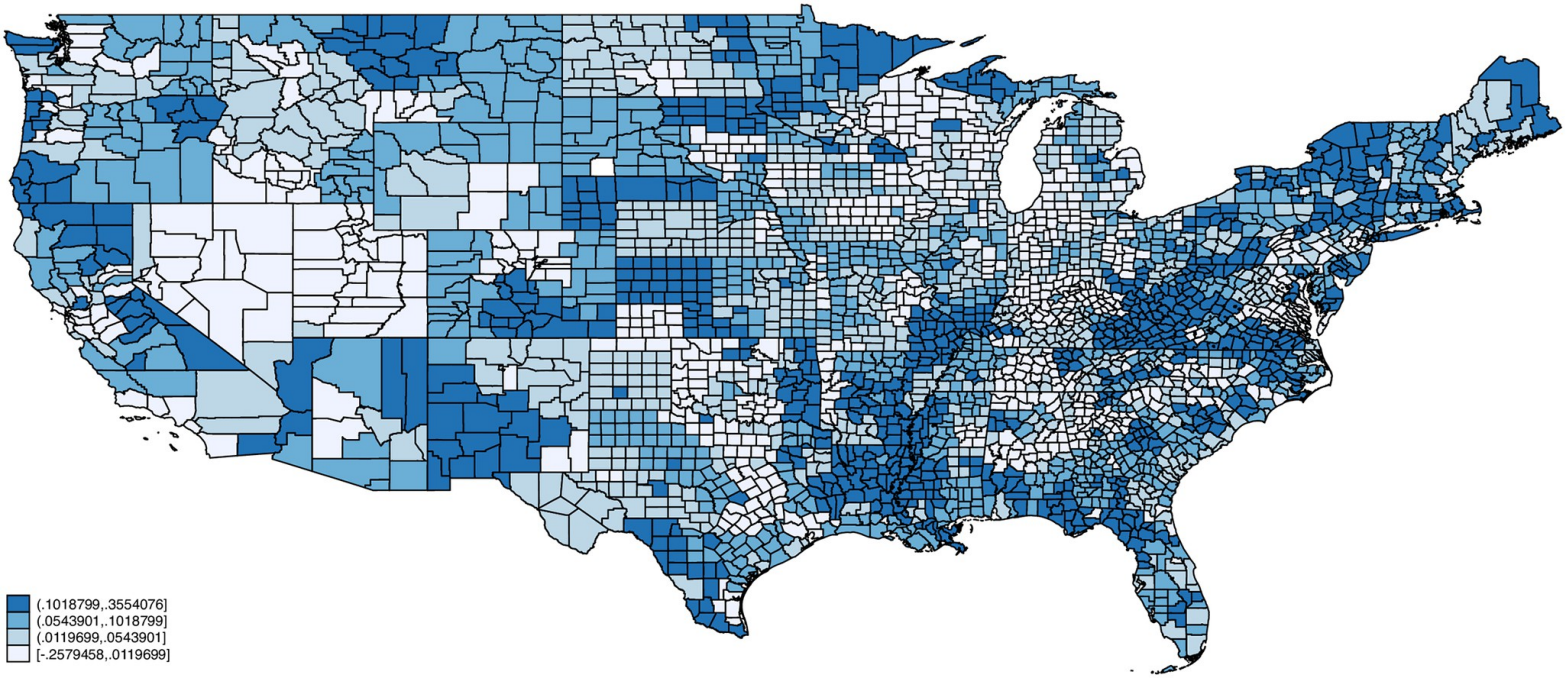
Exposure to infection/disease by county. Notes: Each county is assigned the average value of the exposure index based on the distribution of employment across occupations using 2018 American Community Survey (ACS) data. Endpoints correspond to quartiles. Person weights from the ACS are used.

**Fig 3:**
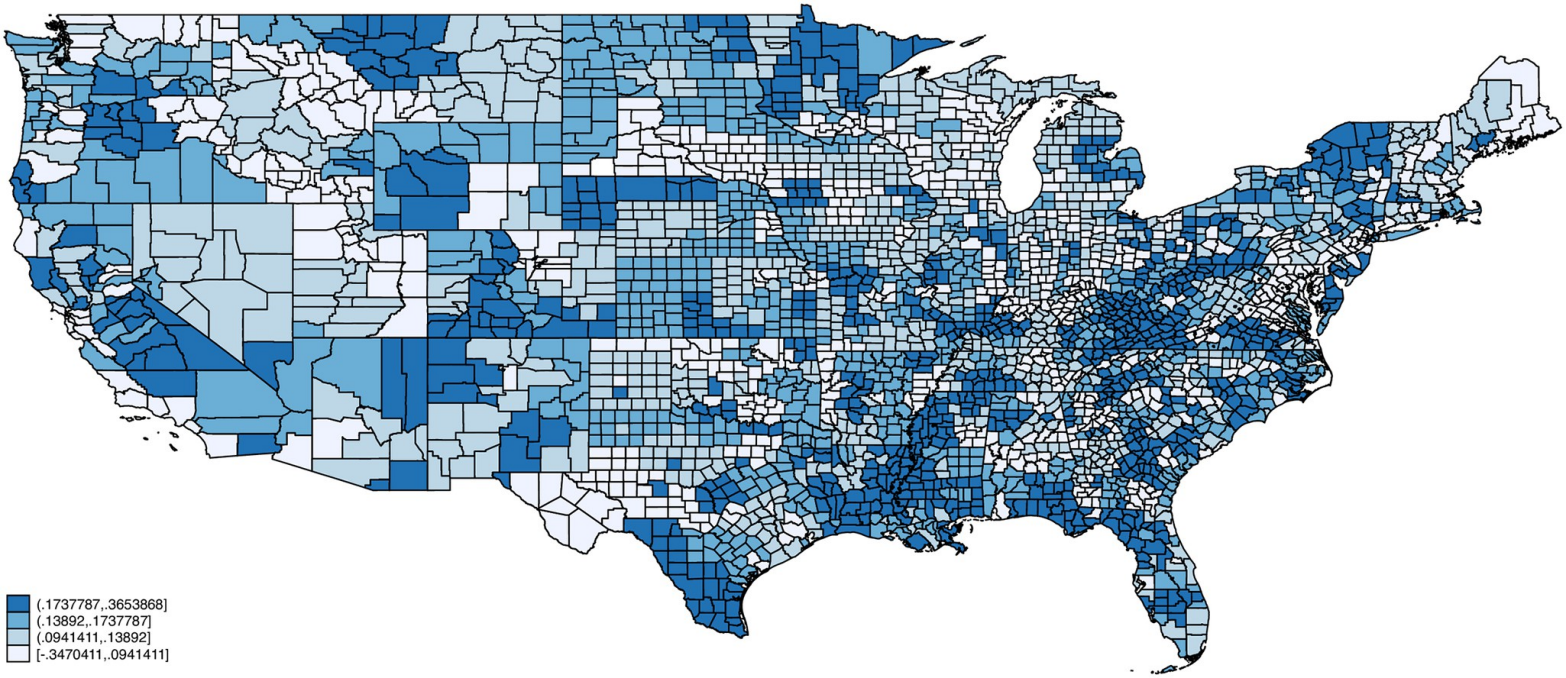
Proximity to coworkers by county. Notes: Each county is assigned the average value of the proximity index based on the distribution of employment across occupations using 2018 American Community Survey (ACS) data. Endpoints correspond to quartiles. Person weights from the ACS are used.

**Fig 4:**
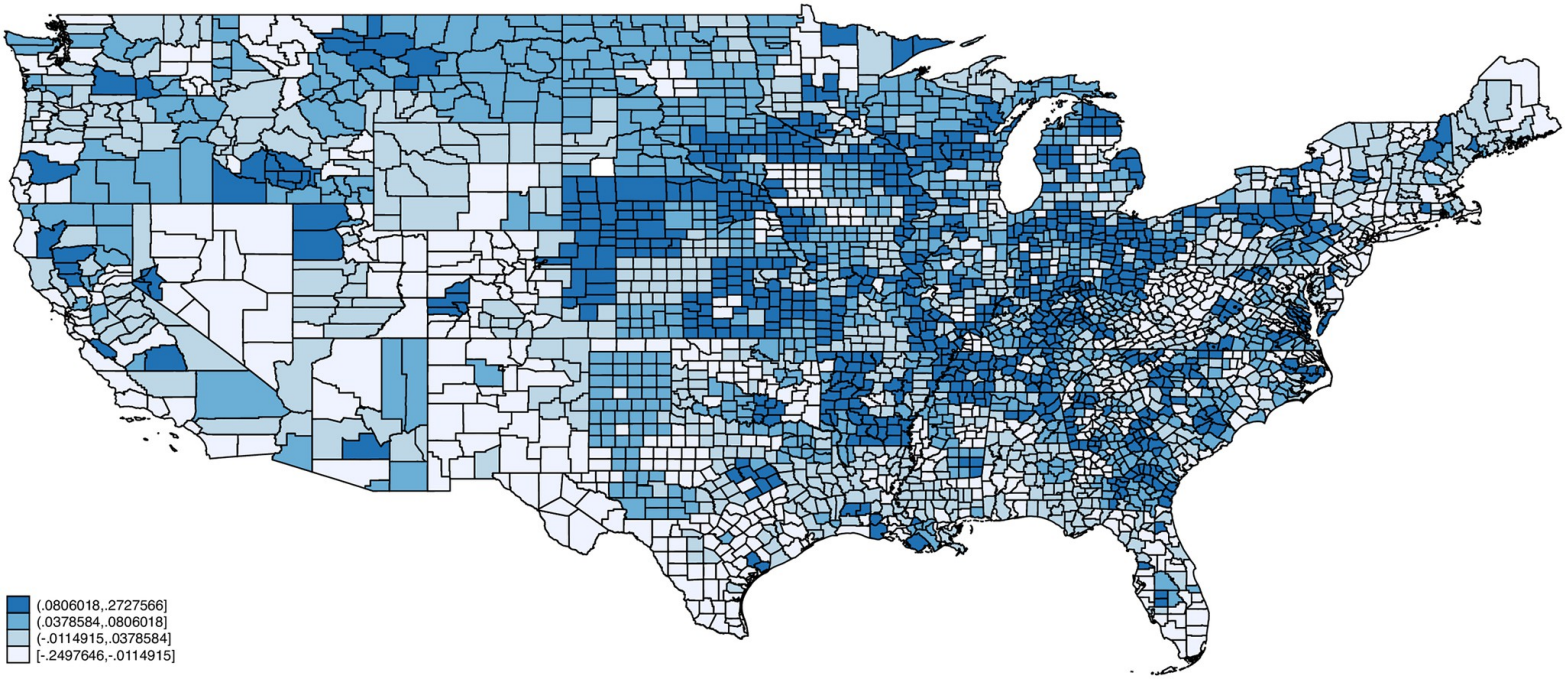
Essential workers by county. Notes: Each county is assigned the average value of the critical index based on the distribution of employment across occupations using 2018 American Community Survey (ACS) data. Endpoints correspond to quartiles. Person weights from the ACS are used.

**Fig 5:**
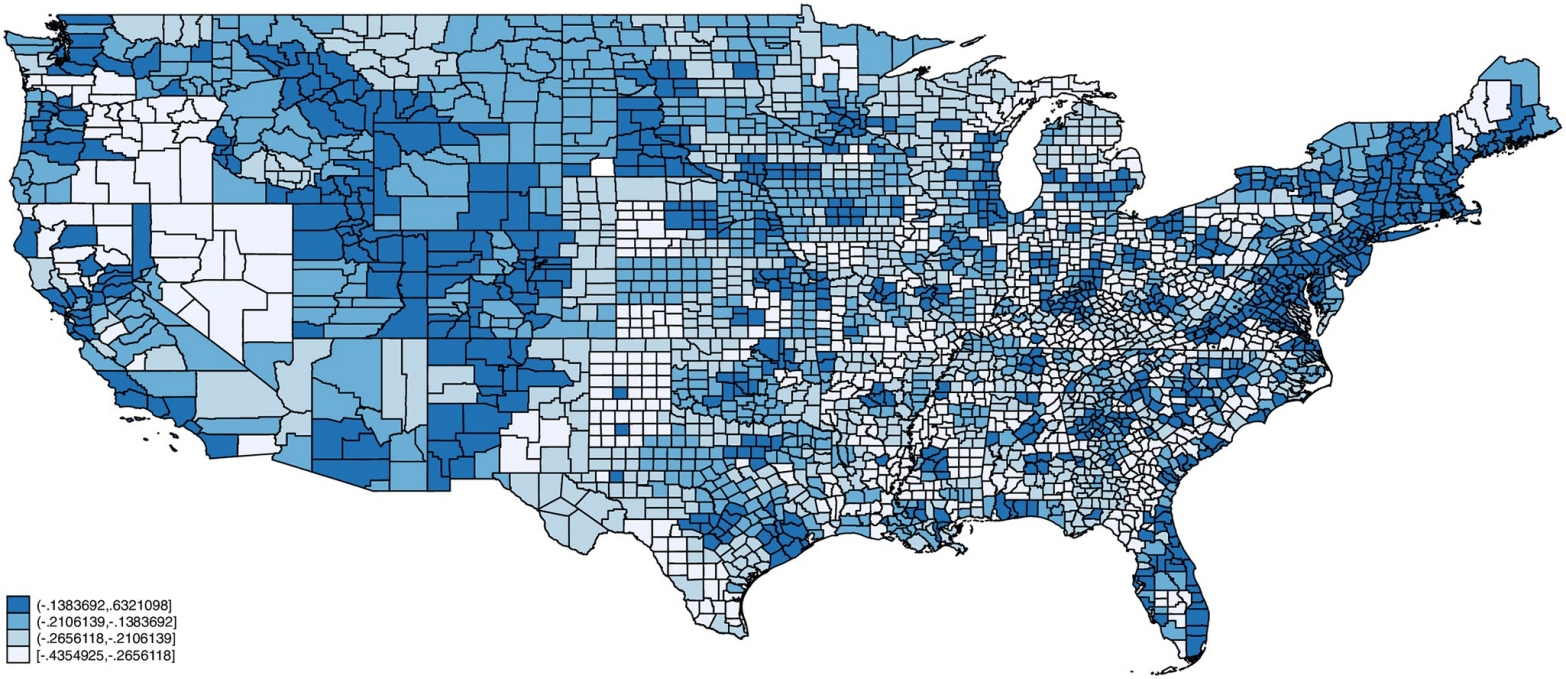
Remote workers by county. Notes: Each county is assigned the average value of the remote worker index based on the distribution of employment across occupations using 2018 American Community Survey (ACS) data. Endpoints correspond to quartiles. Person weights from the ACS are used.

Figs 2–5 plot maps of our four indexes by county. Each county is assigned the average value of the index based on the distribution of employment across occupations using 2018 American Community Survey data. Each map is colored based on quartiles with the lightest color corresponding to index values below the 1st quartile, the next lightest corresponding to values between the 1st and 2nd quartiles, the second darkest corresponding to values between the 2nd and 3rd quartiles, and the darkest corresponding to index values above the 3rd quartile. Exposure to infection and disease, and proximity to coworkers follow similar patterns across space, with concentrations of high exposure and proximity in counties in California, Texas, Florida, Louisiana, and various North and South Eastern states. Critical workers tend to be more concentrated in the Midwestern United States including Wisconsin, Ohio, Nebraska, and Minnesota. Remote Work on the other hand is concentrated in the Northeastern United States as well as parts of Southwest (e.g., New Mexico), West (e.g., Colorado, Utah), and Midwest (e.g., South Dakota).

As we claim that our indexes are useful in explaining why particular subgroups and occupations are more or less affected by COVID-19, it is important to provide descriptions of which people and occupations are exposed to disease, work closely with others, are able to work remotely, and are working in essential occupations. Tables 4 and 5 present the mean values (standard deviations in parentheses) of our four indexes across major occupational classifications (2-digit level) and various demographic and socioeconomic characteristics, respectively.

**Table 4:** Mean index values by occupations.

	Exposure	Proximity	Remote Work	Essential Work
	(1)	(2)	(3)	(4)
Management	-0.501	-0.604	0.749	0.082
	(0.309)	(0.479)	(0.836)	(0.998)
Business/Finance	-0.770	-0.878	1.007	-0.814
	(0.230)	(0.414)	(0.665)	(0.973)
Computer/Mathematical	-0.704	-1.040	1.301	0.083
	(0.337)	(0.699)	(0.000)	(0.998)
Architecture/Engineering	-0.719	-0.640	0.467	0.257
	(0.139)	(0.220)	(0.767)	(0.805)
Physical/Social Science	-0.297	-0.716	0.546	-0.335
	(0.562)	(0.450)	(0.899)	(0.968)
Social Services	0.859	-0.124	0.287	-0.307
	(0.486)	(0.737)	(0.951)	(0.903)
Legal	-0.495	-1.383	1.301	-1.479
	(0.237)	(0.310)	(0.000)	(0.000)
Education	0.429	0.594	1.284	0.658
	(0.782)	(0.808)	(0.078)	(0.232)
Entertainment/Media	-0.640	-0.252	0.750	-1.170
	(0.423)	(0.763)	(0.668)	(0.727)
Healthcare Practitioners	2.497	1.315	-0.728	0.293
	(0.802)	(0.904)	(0.420)	(0.755)
Healthcare Support	2.138	1.343	-0.824	0.539
	(0.813)	(1.069)	(0.230)	(0.523)
Protective Service	0.935	0.584	-0.751	0.553
	(1.039)	(0.664)	(0.387)	(0.546)
Food Preparation/Serving	-0.291	0.893	-0.849	0.082
	(0.300)	(0.370)	(0.000)	(0.998)
Building Cleaning	0.479	-0.850	-0.849	-0.229
	(0.667)	(1.028)	(0.000)	(1.055)
Personal Care and Service	0.738	1.058	-0.154	-1.465
	(0.695)	(0.902)	(0.922)	(0.172)
Sales	-0.339	0.204	-0.291	-0.482
	(0.375)	(0.544)	(0.917)	(1.064)
Office/Admin	-0.047	-0.415	0.553	0.123
	(0.610)	(0.852)	(0.891)	(0.923)
Farming/Fishing/Forestry	-0.624	-1.143	-0.823	0.432
	(0.249)	(0.931)	(0.119)	(0.697)
Construction	-0.449	0.584	-0.847	0.206
	(0.380)	(0.554)	(0.030)	(0.926)
Maintenance	-0.253	-0.116	-0.831	0.576
	(0.510)	(0.542)	(0.198)	(0.499)
Production	-0.608	-0.294	-0.830	0.550
	(0.465)	(0.814)	(0.139)	(0.584)
Transportation/Moving	-0.373	0.001	-0.808	0.409
	(0.659)	(0.857)	(0.255)	(0.730)

Notes: Data from the Current Population Survey. Standard deviations in parentheses. Exposure, Proximity, Remote Work, and Essential Work correspond to the indexes constructed by the authors. Index values are normalized to a mean 0 and standard deviation of 1.

**Table 5:** Mean index values by socio-economic characteristics.

	Exposure	Proximity	Remote Work	Essential Work
	(1)	(2)	(3)	(4)
Male	-0.264	-0.110	-0.135	0.053
	(0.785)	(0.918)	(0.960)	(0.985)
Female	0.293	0.139	0.137	-0.053
	(1.130)	(1.076)	(1.020)	(1.014)
Female with Children	0.364	0.150	0.156	-0.005
	(1.170)	(1.099)	(1.024)	(0.997)
Female without Children	0.233	0.130	0.122	-0.093
	(1.092)	(1.056)	(1.016)	(1.025)
Married	-0.010	-0.072	0.110	-0.001
	(1.029)	(1.003)	(1.016)	(0.999)
Not Married	0.008	0.098	-0.134	0.007
	(0.972)	(0.996)	(0.961)	(1.001)
16–34	0.009	0.134	-0.145	0.007
	(0.981)	(0.992)	(0.956)	(1.001)
35–54	-0.002	-0.049	0.071	0.019
	(1.023)	(1.005)	(1.012)	(0.993)
55 +	-0.018	-0.099	0.088	-0.039
	(0.996)	(0.995)	(1.015)	(1.011)
Asian	-0.018	-0.064	0.108	-0.081
	(1.092)	(1.105)	(1.011)	(1.026)
Black	0.180	0.143	-0.163	0.109
	(1.067)	(1.034)	(0.951)	(0.953)
Hispanic	-0.047	0.071	-0.288	0.067
	(0.899)	(0.981)	(0.901)	(0.980)
Other non-white	-0.003	0.092	-0.175	0.035
	(0.945)	(0.995)	(0.946)	(0.991)
White	-0.029	-0.013	0.020	-0.010
	(0.984)	(0.988)	(1.004)	(1.005)
HS or less	-0.145	0.145	-0.641	0.159
	(0.730)	(0.954)	(0.596)	(0.943)
HS, some college	-0.071	0.092	-0.287	0.065
	(0.867)	(0.958)	(0.896)	(0.979)
College	0.089	-0.095	0.370	-0.083
	(1.144)	(1.042)	(1.002)	(1.021)

Notes: Data from the Current Population Survey. Standard deviations in parentheses. Exposure, Proximity, Remote Work, and Essential Work correspond to the indexes constructed by the authors. Index values are normalized to a mean 0 and standard deviation of 1.

We find significant variation across occupations for our four indexes. We first confirm [1]’s findings that workers in computer, education, law, finance and management are relatively more able to work from home than workers in healthcare, farming, construction and transportation.

Healthcare practitioners, those working in healthcare support, personal care and service, food preparation, and education work more closely with others while legal, farming, computer and mathematical, and business workers work less closely with others. Similarly, those healthcare practitioners and support workers are far and away the most exposed to disease, followed by protective and social services, and personal care workers. Workers who are least exposed to disease come from occupations in business and finance, computer and maths, physical and social sciences, and entertainment and media.

Of note, our analysis does not tackle exposure and proximity outside of the workplace, which could be related to neighborhood characteristics and occupations.

Last, workers in education, healthcare support, protective service, maintenance, production and transportation are more likely to work in essential occupations, whereas workers in law, personal care and entertainment were the less likely.

What emerges from Table 5 is that men are less exposed to disease, work less closely with others, are less able to work remotely and are more likely to work in essential occupations than are women. Women with children are very similar to women without children in terms of working remotely or closely with others but are more often exposed to disease and more likely to work in essential occupations. Younger workers (those aged 16–34) are more exposed to disease, work more closely with others, less able to work remotely than are mid-aged (34–54) or older workers (over 55). Younger workers are however more likely than older workers but less likely than mid-aged workers to work in essential occupations. Among our racial categories, Blacks are most exposed to disease and most likely to work closely with others. Asians are most able to work remotely, followed by Whites while Blacks, other non-white minorities, and Hispanics (respectively) are must less able to work remotely. Blacks, Hispanics, and other non-white minorities are (in that order) the most likely to work in essential occupations while Asians are the least likely. Less educated workers (those with less than high school education) are less exposed to disease, more likely to work closely with others, much less able to work remotely, and more likely to work in essential occupations than are more educated (high school degree, some college completed) and highly educated workers (college degree) workers.

### Occupational measures, wages and unemployment

Figure S5 in S1 Appendix presents the average wage rate from March through June 2020 (i.e., post-COVID-19) by major occupations and our index values. The x-axis indicates the index value while the y-axis indicates the average real hourly wage rate (in 2018 dollars). Each dot is a major occupation category (a two digit SOC code). Panel (a) corresponds to our Exposure Index and indicates that there is not a particularly strong relationship between wages and occupational exposure to disease (R^2^ of 0.011). Panel (b) documents the negative and still relatively weak relationship between wages and proximity to coworkers (R^2^ of 0.081). Panel (c) demonstrates that among these index-wage relationships, it is the remote worker index that is most strongly (and positively) associated with wage rate (R^2^ of 0.262). Panel (d) informs us that there is essentially no relationship between essential workers and the wage rate. To summarize these figures, there’s not much of a relationship between exposure or essential workers and wages, but those workers in occupations that work closely with others tend to have lower wages after COVID-19 while those in occupations able to work remotely tend to have higher wages.

**Fig 6:**
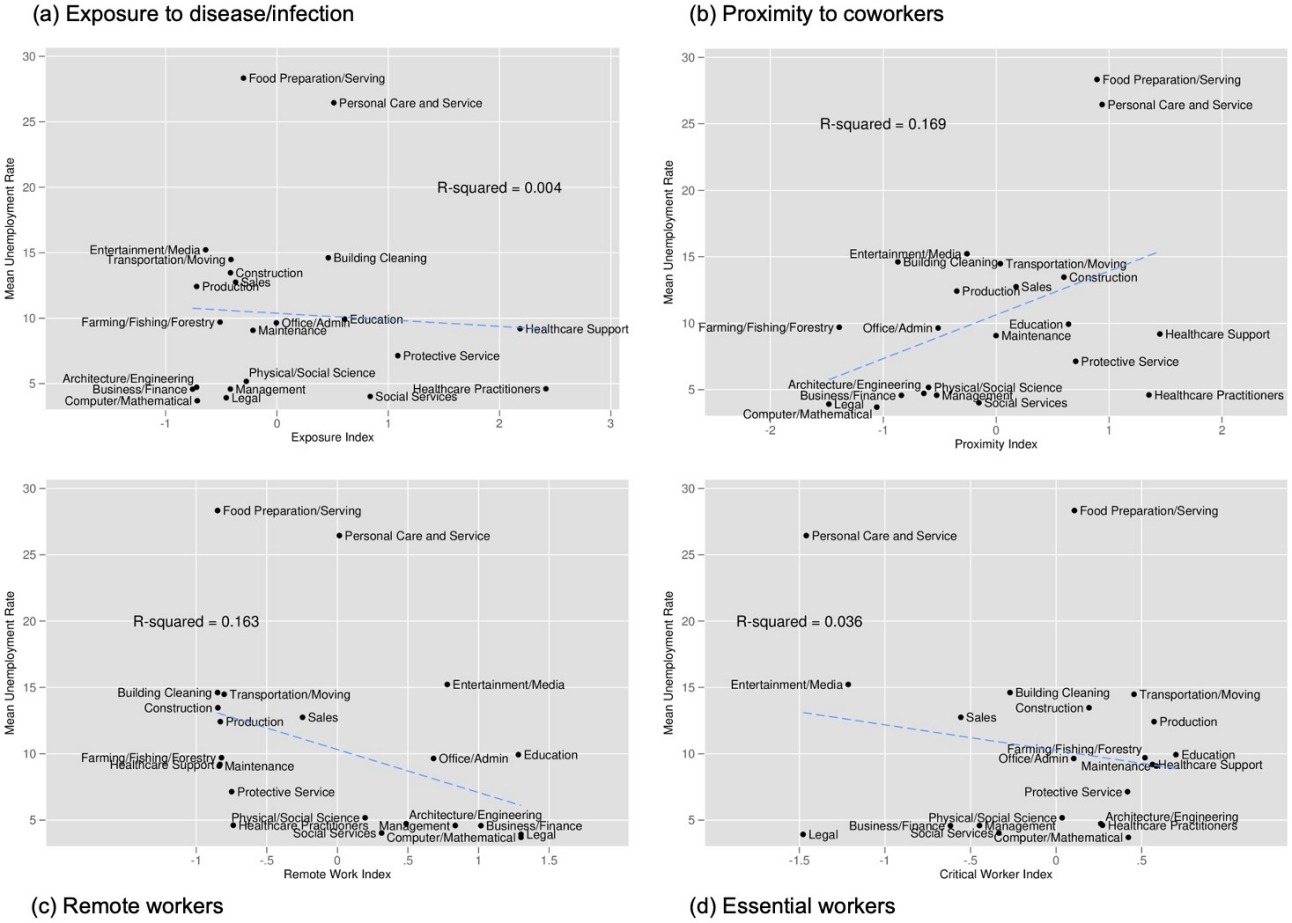
Unemployment by index and occupation. Notes: Unemployment data are from Current Population Survey, indexes are calculated by authors using data from O*NET, LMI, and [1]. The x-axis indicates the index value while the y-axis indicates the average unemployment rate from March 2020 through June 2020. Each dot is a major occupation category (a two digit SOC code). Index values are normalized to mean 0 and standard deviation 1.

Fig 6 shows the analogous figure for the unemployment rate over the same period. In Panel (a) we see little evidence of a relationship between exposure to disease and the average unemployment rate (R^2^ of 0.004). Panel (b) however, indicates the presence of a positive and modest relationship between unemployment and proximity to coworkers (R^2^ of 0.169). Remote work, shown in Panel (c) also has a modest but negative relationship with unemployment (R^2^ of 0.163). Panel (d) shows that there is little to no relationship between essential workers and unemployment (R^2^ of 0.036). In sum, those essential workers and those in occupations more exposed to disease do not tend to have higher or lower unemployment rate at the major occupation level. However, we do see that occupations where workers are more able to work remotely have lower unemployment rates while those occupations where workers are in close proximity to others tend to have higher unemployment rates.

### Empirical strategy

We first rely on a simple pre/post analysis at the national-level. The model is:

$$\begin{array}{c} Y_{i,s,t}=\alpha +\beta PostCOVID_{t}+X_{i,s,t}^{\prime }\gamma +\theta_{s}+\delta_{t}+\varepsilon_{i,s,t} \end{array}$$ (1)

where *Y*_*i*,*s*,*t*_ is an economic outcome for individual *i* in state *s* and month *t*. Our four main outcomes variables are the (1) unemployment rate, (2) labor force participation, (3) hours of work, and (4) hourly wages. Individuals in the labor force were at work; held a job but were temporarily absent from work due to factors like vacation or illness; were seeking work; or were temporarily laid off from a job during the reference period. Hours of work are computed for civilians aged 16–70 who are employed and either at work or absent from work during the survey week, all jobs. Hours of work is trimmed to exclude values below 1st percentile and above 99th percentile. The hourly wages (in 2018 constant dollars) is computed for civilians aged 16–70 currently employed as wage/salary workers, paid hourly, and were in outgoing rotation groups. It excludes self-employed persons and we trim to exclude values below 1st percentile and above 99th percentile.

*Post* *COVID*_*t*_ is an indicator equals to one for March–June 2020 and zero for all preceding months. The time period is January 2016 to June 2020. *X*_*i*,*s*,*t*_ is a vector of other regressors including age, gender, marital status and race. Finally, *θ*_*s*_ and *δ*_*t*_ represent state and time fixed effects, respectively.

We also control for demographic characteristics, the educational level of the respondent and interview type fixed effects, i.e., telephone or in-person. Moreover, to allow for common regional shocks to a given economic outcome, we include interactions between year fixed effects and the four Census regions. We report standard errors clustered at the state-level.

To investigate the effect of indexes on labour market outcomes after COVID-19, we use this modified equation:

$$\begin{array}{c} Y_{i,s,t}=\alpha +\beta_{1}PostCOVID_{t}+\beta_{2}Index_{it}+\beta_{3}Index_{it}*PostCOVID_{t}+X_{i,s,t}^{\prime }\gamma +\theta_{s}+\delta_{t}+\varepsilon_{i,s,t} \end{array}$$ (2)

We add *Index*_*it*_ and *Index*_*it*_ * *Post* *COVID*_*t*_ to Eq 1. *Index*_*it*_ represents the value for the worker for our index of interest (workers able to work remotely; workers relatively more exposed to disease, workers that work with proximity to coworkers and essential/critical workers, as defined above). As described above, the Indexes are standardized to have a mean zero and standard deviation of 1. The coefficient of interest is *β*_3_ and measure the differential impact of the *Index*_*it*_ on the labour market outcome of interest after COVID-19. In the S1 Appendix, as per our PAP, we also replace *Post* *COVID*_*t*_ with measures of case and death rates at the state-level to exploit variation there. The rest of the variables are defined as before.

Lastly, we examine the heterogeneous effects of state-level lockdowns, which we take to mean policies aimed at the suspending of economic activity and social interaction in efforts to reduce COVID-19 transmission, by estimating (we also estimate the effects of lockdowns on our four labor market outcomes using synthetic control methods. See our PAP for more details. The results are in line with our difference-in-differences estimate and suggest that stay-at-home orders increased the unemployment rate by about 3.5 percentage points. Estimates available upon request):

$$\begin{array}{c} Y_{i,s,t}=\alpha +\kappa LOCKDOWN_{s,t}+\beta_{2}Index_{it}+\beta_{3}Index_{it}*LOCKDOWN_{s,t}+X_{i,s,t}^{\prime }\gamma +\theta_{s}+\delta_{t}+\varepsilon_{i,s,t} \end{array}$$ (3)

where *LOCKDOWN* is a binary variable that equals 1 if a state *s* implemented a lockdown, as measured by the announcement of a stay-at-home order. We use data on stay-at-home order dates from the New York Times (see https://www.nytimes.com/interactive/2020/us/coronavirus-stay-at-home-order.html). As per our PAP we include the same suite of demographic controls noted in Eq 1 plus the number of COVID-19 tests performed per 10,000 inhabitants and all other variables are defined as above. Standard errors are clustered at the state-level.

We also include other COVID-19 related controls drawn from [30]: implementation of mandatory face mask policies, day care and school closures, and freezes on eviction and utilities. These additional controls were not pre-specified in our PAP due to data availability at the time of registering.

## Results

In this section, we first describe the relationship between COVID-19 and labor market outcomes, with a particular focus on the different indexes. We then investigate the differential effects of state lockdowns across our indexes.

### Impacts of the pandemic on labor market outcomes

Table 6 presents the OLS estimates of Eq (1) for our four labor market outcome variables as pre-specified in our PAP. The time period is January 2016 to June 2020. The dependent variables are respectively the probability of unemployment, probability of labor force participation, hours of work and hourly wages (Panel (d)). We report standard errors clustered by state. We see a substantial increase in the probability of unemployment and decrease in the probability of labor force participation and hours of work. In contrast, the estimates for hourly wages are positive.

**Table 6:** The impacts of COVID-19: Baseline.

	Unemployed	LFP	Wages	Hours Worked
Post COVID	0.0259	-0.0155	0.0415	-0.4978
	(0.0041)	(0.0024)	(0.1904)	(0.0933)
Observations	3158372	4506713	374408	2865884
Indiv. Chars	Yes	Yes	Yes	Yes
State FE	Yes	Yes	Yes	Yes
Region × Year FE	Yes	Yes	Yes	Yes
Month FE	Yes	Yes	Yes	Yes
Year FE	Yes	Yes	Yes	Yes
Interview Type FE	Yes	Yes	Yes	Yes
State COVID-19 Controls	Yes	Yes	Yes	Yes

Notes: Data from the Current Population Survey. Robust standard errors are in parentheses, adjusted for clustering by state. In column 1 the dependent variable is a dummy for whether the individual is unemployed. In column 2 the dependent variable is a dummy for whether the individual is in the labor force; were at work; held a job but were temporarily absent from work due to factors like vacation or illness; were seeking work; or were temporarily laid off from a job during the reference period. In the third column, the dependent variable is the hourly wages for individuals currently employed as wage/salary workers, paid hourly, and were in outgoing rotation groups. In the fourth column, the dependent variable is hours of work for individuals who are employed and either at work or absent from work during the survey week, all jobs. *Post* *COVID* is a dummy that is equal to one for the months after March 2020. All columns include state, month, year, interview type and Census region × year fixed effects; the following demographic controls: gender, age, marital status, education and race; and the following state COVID-19 related controls: the number of COVID-19 tests performed per 10,000 inhabitants, implementation of mandatory face mask policies, social distancing measures, day care and school closures, and freezes on eviction and utilities. The time period is January 2016–June 2020.

Table 7 provides a similar analysis, interacting the *Post* *COVID* indicator with indicators for April, May, and June. We see a higher probability of unemployment and lower labor force participation and hours early on in the pandemic. The large but transitory increase in wages is consistent with changes in the composition of the labor force (i.e., those who lose their jobs are more likely to come from the lower tail of the wage distribution, increasing average wages mechanically), but we cannot dismiss increased wages through “danger pay” as another mechanism.

**Table 7:** The impacts of COVID-19: Interacted.

	Unemployed	LFP	Wages	Hours Worked
Post COVID × April	0.1159	-0.0376	0.6730	-1.0309
	(0.0074)	(0.0075)	(0.3108)	(0.2737)
Post COVID × May	0.0947	-0.0294	0.1357	-0.9779
	(0.0084)	(0.0077)	(0.3603)	(0.2652)
Post COVID × June	0.0581	-0.0209	0.0049	-0.5754
	(0.0102)	(0.0113)	(0.4755)	(0.2852)
Observations	3158372	4506713	374408	2865884
Indiv. Chars	Yes	Yes	Yes	Yes
State FE	Yes	Yes	Yes	Yes
Region × Year FE	Yes	Yes	Yes	Yes
Month FE	Yes	Yes	Yes	Yes
Year FE	Yes	Yes	Yes	Yes
Interview Type FE	Yes	Yes	Yes	Yes
State COVID-19 Controls	Yes	Yes	Yes	Yes

Notes: Data from the Current Population Survey. Robust standard errors are in parentheses, adjusted for clustering by state. In column 1 the dependent variable is a dummy for whether the individual is unemployed. In column 2 the dependent variable is a dummy for whether the individual is in the labor force; were at work; held a job but were temporarily absent from work due to factors like vacation or illness; were seeking work; or were temporarily laid off from a job during the reference period. In the third column, the dependent variable is the hourly wages for individuals currently employed as wage/salary workers, paid hourly, and were in outgoing rotation groups. In the fourth column, the dependent variable is hours of work for individuals who are employed and either at work or absent from work during the survey week, all jobs. *Post* *COVID* is a dummy that is equal to one for the months after March 2020. All columns include state, month, year, interview type and Census region × year fixed effects; the following demographic controls: gender, age, marital status, education and race; and the following state COVID-19 related controls: the number of COVID-19 tests performed per 10,000 inhabitants, implementation of mandatory face mask policies, social distancing measures, day care and school closures, and freezes on eviction and utilities. The time period is January 2016–June 2020.

Beginning in March 2020, respondents who did not work during the reference week were asked a follow-up question inquiring about the reason for not working. Those who indicated they did not work because they were ill, self-isolating due to health concerns, or were under quarantine were coded as not working due to “own illness, injury, or medical problem” while those who were not ill or quarantined but were not working as a result of the coronavirus were coded as “on layoff” (either temporary or indefinite). If the respondent was uncertain of their return to work within 6 months (the threshold for temporary layoff) interviewers were advised to include them as temporary layoffs.

Respondents who usually worked full-time (35 or more hours) but answered between 1 and 34 hours in the reference week were also asked a follow-up question inquiring about the reason for the change in hours. Those who indicated they did not work because of illness, self-isolation, or quarantine were coded as not working full-time due to “own illness, injury, or medical problem” while those whose hours were reduced for non-illness or quarantine reasons were classified as “slack work or business conditions”.

Despite the guidance given to interviewers, the BLS admitted that some people were misclassified as “employed but not at work” instead of as “unemployed on layoff”. In March 2020 there were 6.4 million people classified as employed but not at work, with 2.1 million of these being classified as “other reasons” (non vacation, illness, family obligation, weather, childcare issues, civic/military duty, school, parental leave). The average of estimates for this category from 2016–2019 is roughly 700,000. The BLS explains that they will not attempt to reclassify individuals who were incorrectly coded ([28]). This misclassification biases our estimates for unemployment effects downwards. A back of the envelope calculation treating all workers above the March average from 2016–2019 who have the “other reasons” explanation for work absence as unemployed (about 1.4 million people) results in an approximately 0.9 percentage point increase in the unemployment rate over the ‘officially’ reported figure. Attempts at reclassifying individuals would require assumptions about who exactly was misclassified, assumptions that could introduce large measurement error for subgroup analysis.

Based on the classification scheme and guidance provided by the BLS, we estimate Eq (1) for those who did not work, who were employed but absent, and those who usually work full-time but did not in the reference week. This analysis could not be pre-specified as we could not know about this misclassification issue until the data were released and should be considered exploratory but in keeping with the spirit of answering our research question about the effects of COVID-19 on labor market outcomes. These results are presented in Table 8—column 1. Panel (a) presents the results for COVID-19 related explanations of unemployment and the dependent variable is a dummy that equals 1 if an unemployed individual is coded as being unemployed either due to “own illness, injury, or medical problem” or “on layoff”. We find that these explanations are approximately 52 percentage points more likely in March-June 2020. Panel (b) provides the estimates of explanations for individuals working part-time instead of their usual full-time hours and the dependent variable is a dummy that equals 1 if the explanation for reduced hours is either “own illness, injury, or medical problem” or “slack work or business conditions”. Our estimates suggest the COVID-19 related explanations are roughly 16 percentage points more likely in March-June. Panel (c) contains estimates for the explanations of work absences and the dependent variable is a dummy that equals one if the explanation for being absent is “other reasons”. This COVID-19 related explanation is about 39 percentage points more likely in March-June. These results are statistically significant at the 1% level and suggest that using the unemployment rate as a dependent variable leads to underestimating the economic impacts of COVID-19.

**Table 8:** COVID-19-related absences, layoffs and involuntary part-time: Exposure, proximity, remote and essential work.

*Panel A. Unemployed*					
	Baseline	Exposure	Proximity	Remote	Essential
	(1)	(2)	(3)	(4)	(5)
Post COVID	0.517	0.520	0.508	0.511	0.514
	(0.0114)	(0.0111)	(0.0118)	(0.0108)	(0.0104)
Index		0.000281	0.0221	-0.0212	0.000820
		(0.00257)	(0.00575)	(0.00402)	(0.00347)
Index × Post		0.0386	0.0272	-0.0140	-0.0140
		(0.00551)	(0.00789)	(0.00715)	(0.00458)
Observations	138942	126605	126605	121696	121550
*Panel B. Reduced Hours*					
	Baseline	Exposure	Proximity	Remote	Essential
	(1)	(2)	(3)	(4)	(5)
Post COVID	0.164	0.166	0.165	0.164	0.163
	(0.00776)	(0.00787)	(0.00795)	(0.00744)	(0.00742)
Index		-0.00599	-0.00417	-0.0178	0.00975
		(0.000942)	(0.000846)	(0.00151)	(0.000771)
Index × Post		-0.00914	-0.00379	0.00197	-0.0137
		(0.00330)	(0.00362)	(0.00341)	(0.00373)
Observations	658334	658334	658334	634278	633977
Indiv. Chars	Yes	Yes	Yes	Yes	Yes
State FE	Yes	Yes	Yes	Yes	Yes
Region × Year FE	Yes	Yes	Yes	Yes	Yes
Month FE	Yes	Yes	Yes	Yes	Yes
Year FE	Yes	Yes	Yes	Yes	Yes
Interview Type FE	Yes	Yes	Yes	Yes	Yes
State COVID-19 Controls	Yes	Yes	Yes	Yes	Yes
*Panel C. Absences*					
	Baseline	Exposure	Proximity	Remote	Essential
	(1)	(2)	(3)	(4)	(5)
Post COVID	0.387	0.394	0.385	0.389	0.381
	(0.0215)	(0.0215)	(0.0213)	(0.0213)	(0.0197)
Index		-0.00764	0.00204	0.0147	-0.0108
		(0.00140)	(0.00147)	(0.00192)	(0.00121)
Index × Post		-0.0396	0.00735	-0.0323	-0.0815
		(0.00551)	(0.00759)	(0.00733)	(0.00533)
Observations	109126	109126	109126	105891	105769
Indiv. Chars	Yes	Yes	Yes	Yes	Yes
State FE	Yes	Yes	Yes	Yes	Yes
Region × Year FE	Yes	Yes	Yes	Yes	Yes
Month FE	Yes	Yes	Yes	Yes	Yes
Year FE	Yes	Yes	Yes	Yes	Yes
Interview Type FE	Yes	Yes	Yes	Yes	Yes
State COVID-19 Controls	Yes	Yes	Yes	Yes	Yes

Notes: Data from the Current Population Survey. Robust standard errors are in parentheses, adjusted for clustering by state. In the top panel, the dependent variable is a dummy that equals one if an individual’s explanation for unemployment falls into the BLS advised category for COVID-19 related layoffs. In the second panel, the dependent variable is a dummy that equals one if the individual’s explanation for working part-time hours when usually full-time calls into the BLS advised category for COVID-19 related slack. In the bottom panel, the dependent variable is a dummy that equals one if the individual’s explanation for why they were absent at their job in the reference week falls into the “other” category the BLS identifies as being a location for misclassified workers. In column 1 of all panels, we provide baseline estimates without the indexes. Columns 2–5 provide estimates for our indexes. *Index* measures our exposure to disease index, proximity to coworkers index, remote work index, and essential worker index, respectively. *Post* *COVID* is a dummy that is equal to one for the months after March 2020. All columns include state, month, year, interview type and Census region × year fixed effects and the following demographic controls: gender, age, marital status, education and race; and the following state COVID-19 related controls: the number of COVID-19 tests performed per 10,000 inhabitants, implementation of mandatory face mask policies, social distancing measures, day care and school closures, and freezes on eviction and utilities. The time period is January 2016–June 2020.

#### Impact of the pandemic by occupational tasks

We now explore whether COVID-19 had larger impacts on workers relatively more exposed to disease, working in proximity to coworkers, who can easily work remotely and essential workers.

**Fig 7:**
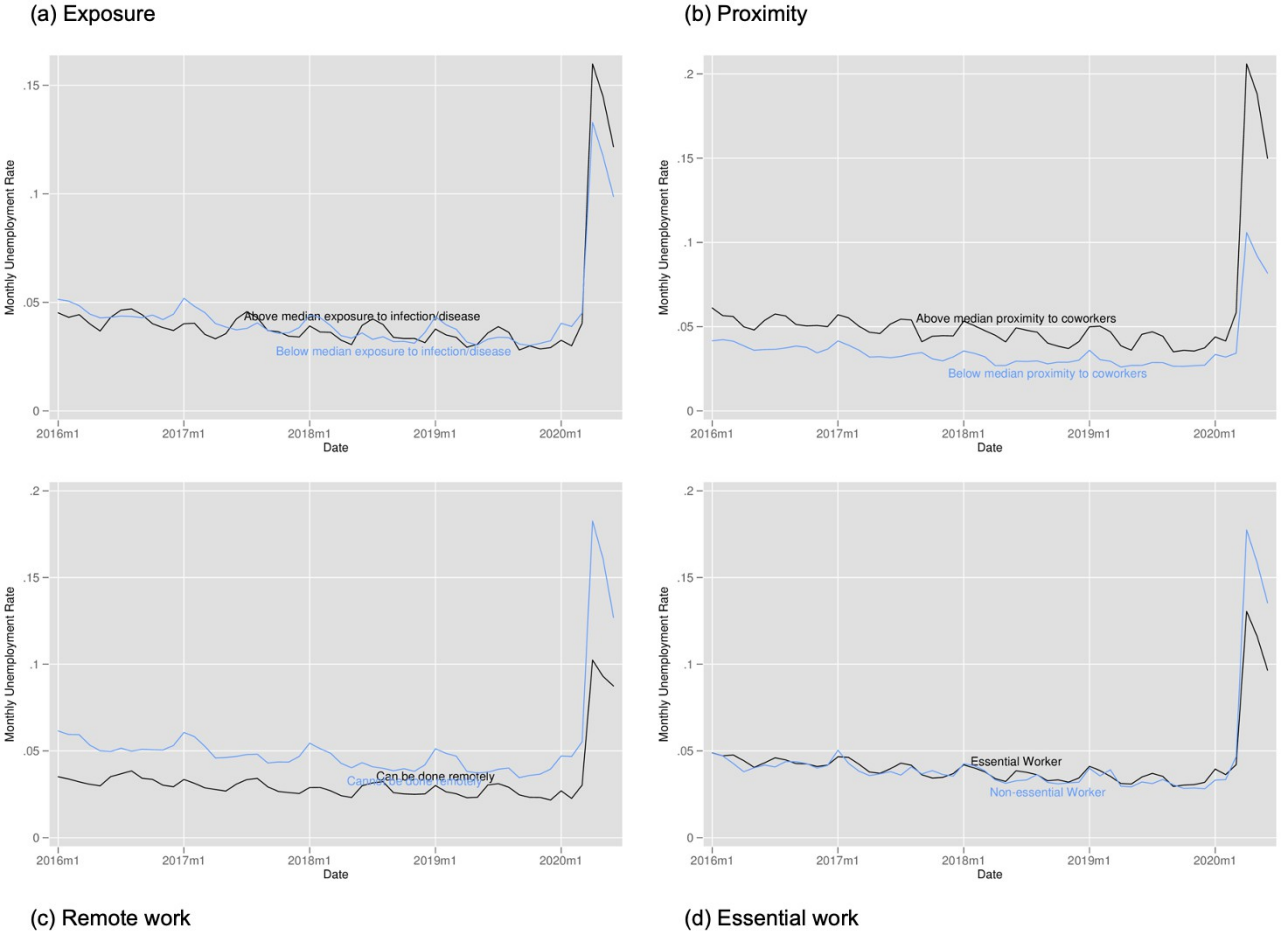
Unemployment rate by exposure to disease, proximity to coworkers, remote work, and essential work. Notes: Authors’ calculations. Data from the Current Population Survey. The time period is January 2016 to June 2020. Each panel plots the unemployment rate. Panel A for individuals in occupations above and below the median for our index of exposure to the disease. Panel B for individuals in occupations above and below the median for our index of proximity to coworkers. Panel C for individuals in occupations designated by [1] as being able to be done remotely. Panel D for individuals in occupations designated as essential and non-essential by the Labor Market Information Institute.

Fig 7 plots the unemployment rate for workers above and below the median values of our exposure to disease and infections index in Panel (a); above and below median values of our proximity to coworkers index in Panel (b); workers whose jobs can and cannot be done remotely in Panel (c); and workers whose jobs are classified as essential or non-essential in Panel (d). This figure suggests that workers in occupations with above median exposure or proximity experienced a slightly more pronounced increase in unemployment than workers with below median exposure or risk. Non-essential workers and those who cannot work remotely saw a smaller increase in unemployment than those who can work remotely or who are considered essential. However, while the recovery appears to be very similar for workers above and below median exposure and for essential and non-essential workers, those below median proximity and who can work remotely saw weaker recoveries than their counterparts.

**Fig 8:**
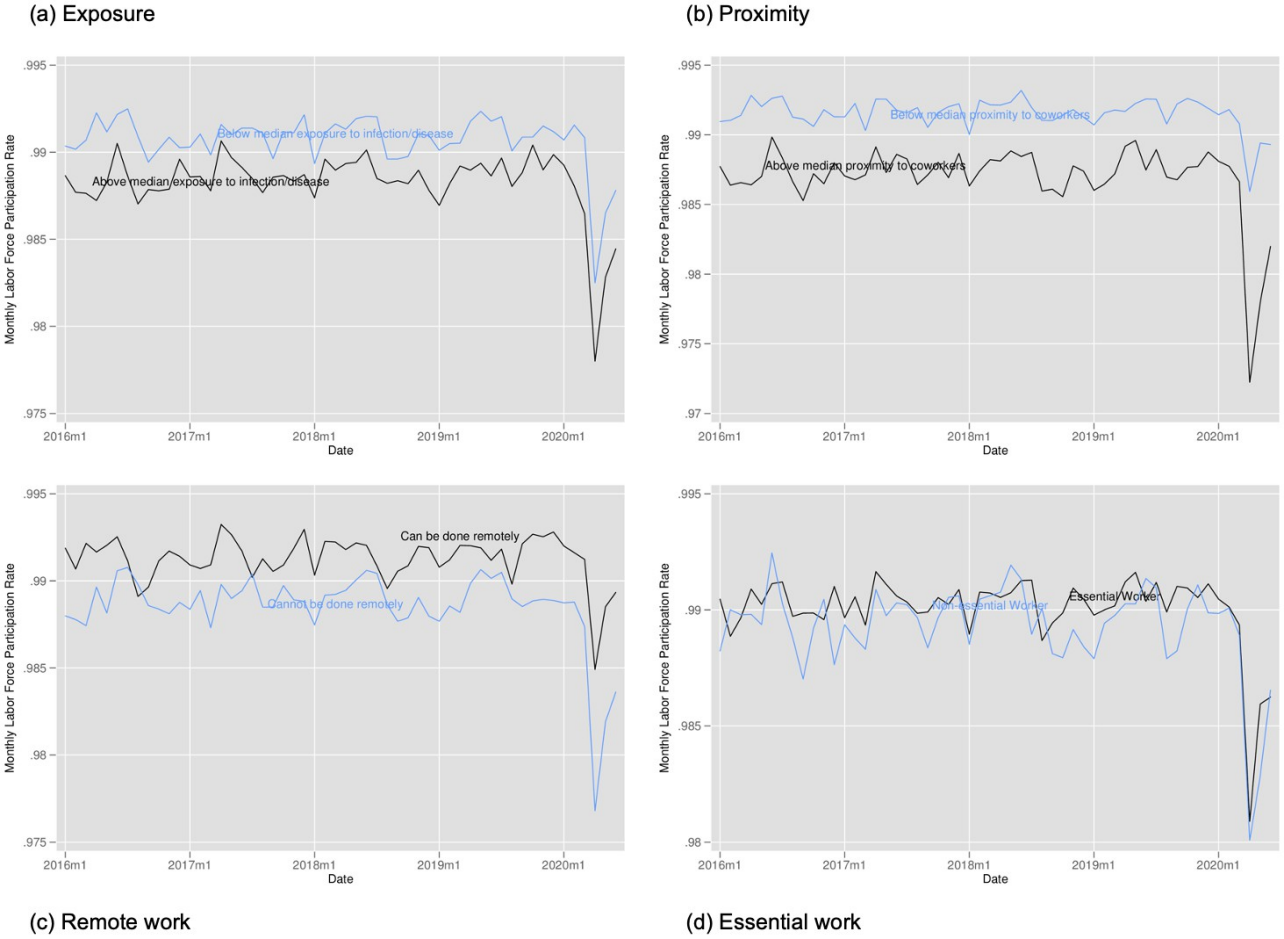
Labor force participation by exposure to disease, proximity to coworkers, remote work, and essential work. Notes: Authors’ calculations. Data from the Current Population Survey. The time period is January 2016 to June 2020. Each panel plots the monthly labor force participation rate. Panel A for individuals in occupations above and below the median for our index of exposure to the disease. Panel B for individuals in occupations above and below the median for our index of proximity to coworkers. Panel C for individuals in occupations designated by [1] as being able to be done remotely. Panel D for individuals in occupations designated as essential and non-essential by the Labor Market Information Institute. Individuals in the labor force were at work; held a job but were temporarily absent from work due to factors like vacation or illness; were seeking work; or were temporarily laid off from a job during the reference period.

**Fig 9:**
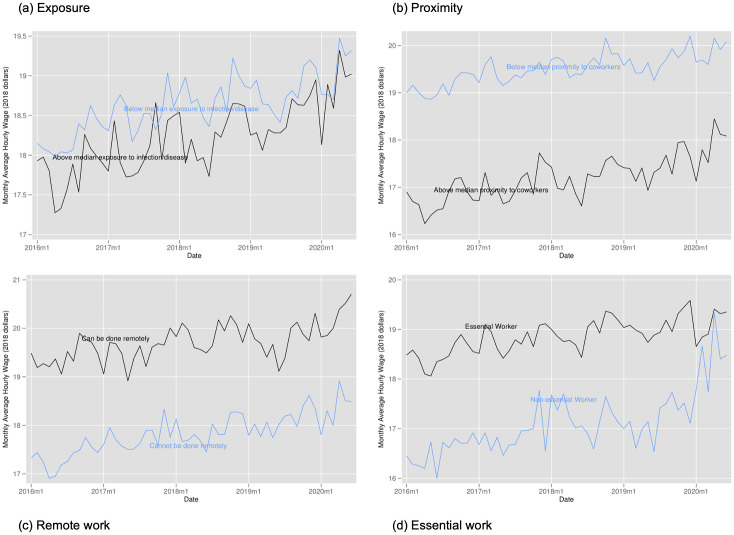
Hourly wages by exposure to disease, proximity to coworkers, remote work, and essential work. Notes: Authors’ calculations. Data from the Current Population Survey. The time period is January 2016 to June 2020. Each panel plots the monthly average hourly wage. Panel A for individuals in occupations above and below the median for our index of exposure to the disease. Panel B for individuals in occupations above and below the median for our index of proximity to coworkers. Panel C for individuals in occupations designated by [1] as being able to be done remotely. Panel D for individuals in occupations designated as essential and non-essential by the Labor Market Information Institute. Hourly wages: civilians aged 16–70 currently employed as wage/salary workers, paid hourly, and were in outgoing rotation groups. Excludes self-employed persons. Trimmed to exclude values below 1st percentile and above 99th percentile. Reported in 2018 constant dollars.

**Fig 10:**
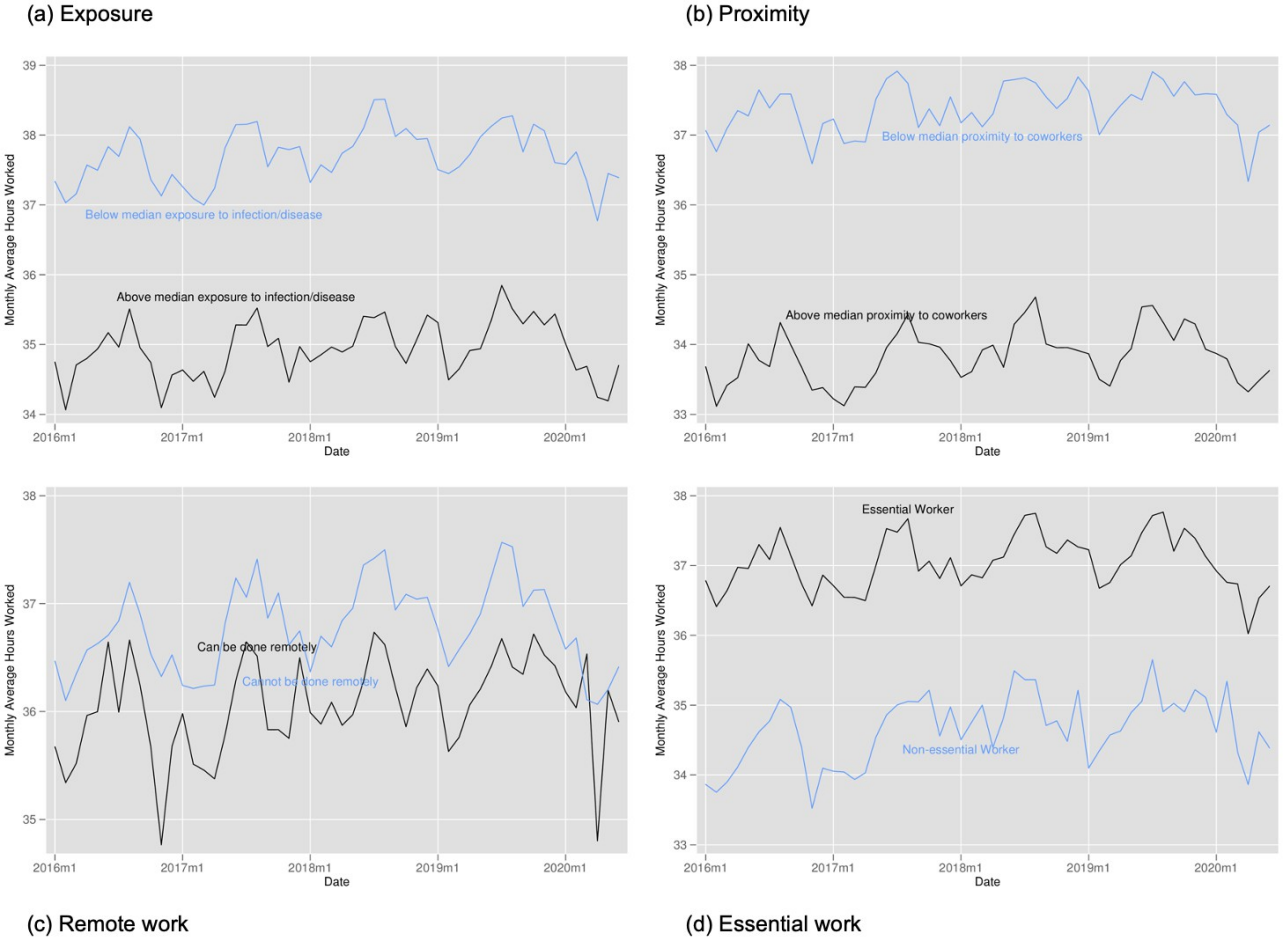
Hours worked by exposure to disease, proximity to coworkers, remote work, and essential work. Notes: Authors’ calculations. Data from the Current Population Survey. The time period is January 2016 to June 2020. Each panel plots the average weekly hours worked. Panel A for individuals in occupations above and below the median for our index of exposure to the disease. Panel B for individuals in occupations above and below the median for our index of proximity to coworkers. Panel C for individuals in occupations designated by [1] as being able to be done remotely. Panel D for individuals in occupations designated as essential and non-essential by the Labor Market Information Institute. Hours worked: civilians aged 16–70 who are employed and either at work or absent from work during the survey week, all jobs. Trimmed to exclude values below 1st percentile and above 99th percentile.

Figs 8–10 provide the analogous tables for labor force participation, hourly wages, and hours of work. Labor force participation falls more steeply for those with above average proximity to coworkers, those with above average exposure, and for those who cannot work remotely but falls very similarly for essential and non-essential workers. We see suggestive evidence that hours of work fall more steeply for more exposed workers, for workers less in proximity to coworkers, and for those more able to work from home (though this quickly rebounds). While we find some evidence of wages increases for non-essential workers and those with above average proximity to coworkers, this likely stems from job losses being concentrated among those with lower wages rather than actual wage increases.

In Table 9, we follow our PAP and formally test whether COVID-19 had differential impacts on our subgroups of workers for our four labor market outcomes of interest. Column 1 presents the estimates for probability of being unemployed, column 2 for the probability of being in the labor force, column 3 for the real hourly wage rate, and column 4 for hours worked. All columns include our full set of fixed effects and demographic controls and include *Post* *COVID*, all four indexes and their interactions with *Post* *COVID* following Eq (2).

**Table 9:** The impacts of COVID-19: Exposure, proximity, remote work and essential workers.

	Unemployed	LFP	Wages	Hours
Post COVID-19	0.0246	-0.0019	0.0830	-0.5322
	(0.0039)	(0.0008)	(0.1799)	(0.1084)
Exposure	-0.0066	0.0004	1.1403	0.1609
	(0.0004)	(0.0001)	(0.0374)	(0.0323)
Exposure × Post	-0.0173	0.0013	-0.1203	0.0135
	(0.0018)	(0.0004)	(0.0965)	(0.0619)
Essential Worker	-0.0004	0.0003	0.4854	0.4105
	(0.0003)	(0.0001)	(0.0249)	(0.0406)
Essential Worker × Post	-0.0203	0.0010	-0.1255	-0.1474
	(0.0018)	(0.0003)	(0.0755)	(0.0577)
Remote Work	-0.0045	-0.0001	0.8392	0.0742
	(0.0004)	(0.0001)	(0.0635)	(0.0234)
Remote Work × Post	-0.0176	0.0015	0.0489	0.0655
	(0.0023)	(0.0004)	(0.1202)	(0.0568)
Proximity	0.0028	-0.0008	-0.5295	-0.9924
	(0.0008)	(0.0001)	(0.0223)	(0.0670)
Proximity × Post	0.0306	-0.0023	0.1308	-0.0620
	(0.0030)	(0.0005)	(0.0736)	(0.0766)
Observations	3028575	3060155	359535	2758846
Indiv. Chars	Yes	Yes	Yes	Yes
State FE	Yes	Yes	Yes	Yes
Interview Type FE	Yes	Yes	Yes	Yes
Region × Year FE	Yes	Yes	Yes	Yes
Month FE	Yes	Yes	Yes	Yes
Year FE	Yes	Yes	Yes	Yes
State COVID-19 Controls	Yes	Yes	Yes	Yes

Notes: Data from the Current Population Survey. Robust standard errors are in parentheses, adjusted for clustering by state. In column 1 the dependent variable is a dummy for whether the individual is unemployed. In column 2 the dependent variable is a dummy for whether the individual is in the labor force; were at work; held a job but were temporarily absent from work due to factors like vacation or illness; were seeking work; or were temporarily laid off from a job during the reference period. In the third column, the dependent variable is the hourly wages for individuals currently employed as wage/salary workers, paid hourly, and were in outgoing rotation groups. In the fourth column, the dependent variable is hours of work for individuals who are employed and either at work or absent from work during the survey week, all jobs. *Exposure* measures our exposure to disease index, *Proximity* is the proximity to coworkers index, *RemoteWork* is the remote work index, and *EssentialWorker* is our essential worker index, respectively. *Post* *COVID* is a dummy that is equal to one for the months after March 2020. All columns include state, month, year, interview type and Census region × year fixed effects; the following demographic controls: gender, age, marital status, education and race; and the following state COVID-19 related controls: the number of COVID-19 tests performed per 10,000 inhabitants, implementation of mandatory face mask policies, social distancing measures, day care and school closures, and freezes on eviction and utilities. The time period is January 2016–June 2020.

In column 1, we first note that after COVID-19 emerges, everyone is more likely to be unemployed. We can also see that in general, workers who are more able to work remotely and those who are more exposed to disease are less likely to be unemployed than those who are not as able to work remotely or who are not as exposed. Conversely, those who work more closely in proximity to others are more likely to be unemployed than those you work farther from others. We also find that workers more likely to be deemed essential do not appear to be more or less likely to be employed than their counterparts. Moving on to the main coefficients of interest, the interactions terms, we see that after COVID-19 workers who are more exposed to disease are 1.7 percentage points less likely to be unemployed than workers who are less exposed, a 69% reduction in the baseline post-COVID-19 probability. Workers who work more closely to others are 3 percentage points more likely to be unemployed than those who work farther from others, which is 124% of the baseline probability. After COVID-19 appears, those more able to work remotely are 1.7 percentage points less likely to be unemployed, about 69% as large as the baseline probability. Lastly, workers more likely to be essential are 2 percentage points less likely to be unemployed, about 83% as large as the post-COVID baseline. These results are all statistically significant at the 1% level.

In column 2, we note that the post COVID baseline reduction in the probability of labor force participation is 0.2 percentage points lower. In general, workers who are more exposed to disease and those who are more likely to be essential are more likely to be in the labor force while those who work more closely with others and those who can work remotely are less likely to be in the labor force. Post COVID, workers who are more exposed to disease are 0.13 percentage points more likely to be in the labor force than less exposed workers, which is 68% of the baseline reduction. For workers that work more closely with others, they are actually 0.23 percentage points less likely than those working farther from others to be in the labor force (an additional reduction that is 121% the size of the baseline). Workers who are more able to work from home are 0.15 percentage points more likely to be in the labor force than those who are less able to work from home, about 79% as large as the post COVID baseline estimate. For workers more likely to be essential workers, we see a 0.1 percentage point increase in the likelihood that they are in the labor force post COVID compared to those less likely to be essential workers. These estimates are statistically significant at the 1% level.

Column 3 indicates that after COVID’s appearance, wages are slightly higher, but the point estimates are statistically insignificant. We also stress that changes in wages could be driven by job loss concentrated among lower wage workers. Workers who are more exposed, more able to work remotely, and more likely to be essential workers earn more than their counterparts while workers who operate more closely with others earn less than those who work farther away from others. Our interaction term estimates are negative for workers more likely to be essential and more exposed to disease workers but positive for workers in closer proximity and workers more able to work remotely. However, this evidence is not strong as only the proximity index interaction is statistically significant at conventional levels (the 10% level in this case).

Column 4 indicates that post COVID, there was an average reduction of about 0.5 hours per week. While there is less immediate evidence that compositional effects play a large role for hours worked than for hourly wages, we cannot rule out their influence. Generally speaking more exposed workers, workers more likely to be essential and more remote workers work more than their counterparts, though the magnitudes of these differences is not particularly large, economically speaking (0.4 hours per week being the largest). Meanwhile, workers who operate more closely with coworkers generally work about 1 hour a week less than those who work less closely with coworkers. We see that our interaction term point estimates contain both positive and negative values, however only essential workers are statistically significant at conventional levels (again the 10% level in this case) and none of these estimates are especially economically meaningful in magnitude.

Columns 2–5 of Table S3 S1 Appendix, provide a similar analysis where each index is included individually rather than all together. The estimates remain similar in magnitude and direction with the exception of the estimates for the exposure to infection/disease index interaction term which are not statistically significant for unemployment or labor market participation. These kinds of reversals are not present for the exposure index for hours worked nor wages. These results could be explained by our exposure index’s strong positive correlation with our proximity index and negative correlation with our remote work index. That the estimates remain unchanged for proximity and remote work indexes but change for the exposure index might suggest that the labor market effects from COVID-19 only matter to exposed workers who are unable to work remotely and who work closely with others. That is, within occupations, exposure matters but it does not appear to provide much explanatory value across occupations.

A natural question is to what extent the observed estimates for the essential worker index are driven by essential workers in the healthcare field. Table S4 in S1 Appendix provides estimates for such a breakdown but was not pre-specified in our PAP and should be considered exploratory. Column 1 provides the overall effect on essential workers while columns 2 and 3 present results for healthcare and non-healthcare based essential workers, respectively. Non-healthcare based essential workers are less likely to be unemployed than other less essential non-healthcare based workers. This is even more pronounced for healthcare based essential workers. Both healthcare based and non-healthcare based essential workers are neither more nor less likely to participate in the labor force after COVID-19 emerges compared to less essential healthcare and non-healthcare workers. We do see that the change in hours worked seems to be driven by fewer hours for healthcare based essential workers and that wage estimates appear to be driven up by healthcare essential workers, though again we stress that these changes are likely capturing compositional changes in the labor force in addition to any wage premia that may exist.

In Table 10 we study COVID-19 related absences, reduced hours, and unemployment using our indexes and following Eq (2). In columns 2–5 of Table 8 we provide estimates where indexes are included individually which are very similar. In sum, we find that all groups of workers see an increase in unemployment, reduced hours and absences from work related to COVID-19. However, we find heterogeneity across our indexes. We find that workers able to work remotely and essential workers are significantly less likely to report COVID-19 unemployment and absences. In contrast, workers working in proximity to others and workers with more exposure are significantly more likely to report COVID-19 reasons for unemployment. Changes in reduced hours are only statistically significant for essential workers and more exposed workers but the changes in the probability of providing these responses amounts to tenths of a percentage point.

**Table 10:** COVID-19-related absences, layoffs and involuntary part-time: Exposure, proximity, remote and essential work.

	Unemployed	Reduced Hours	Absences
Post COVID-19	0.2709	0.0726	0.1548
	(0.0327)	(0.0097)	(0.0425)
Exposure	-0.0104	-0.0110	-0.0139
	(0.0019)	(0.0008)	(0.0034)
Proximity	0.0263	-0.0057	0.0205
	(0.0075)	(0.0010)	(0.0049)
Remote Work	-0.0075	-0.0194	0.0200
	(0.0039)	(0.0010)	(0.0028)
Essential Worker	-0.0000	0.0079	-0.0071
	(0.0026)	(0.0006)	(0.0026)
Exposure × Post	0.0309	-0.0077	-0.0428
	(0.0049)	(0.0031)	(0.0102)
Proximity × Post	0.0157	0.0040	0.0109
	(0.0085)	(0.0036)	(0.0142)
Remote Work × Post	-0.0101	-0.0004	-0.0266
	(0.0065)	(0.0037)	(0.0102)
Essential Worker × Post	-0.0160	-0.0148	-0.0561
	(0.0044)	(0.0038)	(0.0104)
Observations	121550	633977	27033
Indiv. Chars	Yes	Yes	Yes
State FE	Yes	Yes	Yes
Interview Type FE	Yes	Yes	Yes
Region × Year FE	Yes	Yes	Yes
Month FE	Yes	Yes	Yes
Year FE	Yes	Yes	Yes
State COVID-19 Controls	Yes	Yes	Yes

Notes: Data from the Current Population Survey. Robust standard errors are in parentheses, adjusted for clustering by state. In column 1, the dependent variable is a dummy that equals one if an individual’s explanation for unemployment falls into the BLS advised category for COVID-19 related layoffs. In the second column, the dependent variable is a dummy that equals one if the individual’s explanation for working part-time hours when usually full-time calls into the BLS advised category for COVID-19 related slack. In column 3, the dependent variable is a dummy that equals one if the individual’s explanation for why they were absent at their job in the reference week falls into the “other” category the BLS identifies as being a location for misclassified workers. *Exposure* measures our exposure to disease index, *Proximity* is the proximity to coworkers index, *RemoteWork* is the remote work index, and *EssentialWorker* is our essential worker index, respectively. *Post* *COVID* is a dummy that is equal to one for the months after March 2020. All columns include state, month, year, interview type and Census region × year fixed effects; the following demographic controls: gender, age, marital status, education and race; and the following state COVID-19 related controls: the number of COVID-19 tests performed per 10,000 inhabitants, implementation of mandatory face mask policies, social distancing measures, day care and school closures, and freezes on eviction and utilities. The time period is January 2016–June 2020.

### Impacts of the lockdowns on labor market outcomes

Next, we continue to answer questions detailed in our PAP and turn to Table 11 where we estimate the differential effects of lockdowns across our indexes (Eq 3). We include all indexes and interactions in the specification. We also include other COVID-19 related controls: the number of COVID-19 tests performed per 10,000 inhabitants, implementation of mandatory face mask policies, social distancing measures, day care and school closures, and freezes on eviction and utilities.

**Table 11:** The impacts of lockdowns: Exposure, proximity, remote work and essential workers.

	Unemployed	LFP	Wages	Hours
Exposure	-0.0067	0.0004	1.1414	0.1634
	(0.0004)	(0.0001)	(0.0376)	(0.0326)
Lockdown	0.0683	-0.0081	0.2746	-0.9937
	(0.0166)	(0.0017)	(0.2090)	(0.2359)
Exposure × Lockdown	-0.0222	0.0020	-0.2150	-0.0317
	(0.0023)	(0.0005)	(0.1005)	(0.0697)
Proximity	0.0029	-0.0008	-0.5298	-0.9932
	(0.0008)	(0.0001)	(0.0224)	(0.0666)
Proximity × Lockdown	0.0402	-0.0031	0.2041	-0.0711
	(0.0043)	(0.0007)	(0.1024)	(0.0917)
Remote Work	-0.0046	-0.0001	0.8406	0.0781
	(0.0005)	(0.0001)	(0.0635)	(0.0235)
Remote Work × Lockdown	-0.0233	0.0020	0.0162	0.0218
	(0.0032)	(0.0005)	(0.1860)	(0.0725)
Essential Worker	-0.0005	0.0003	0.4836	0.4083
	(0.0003)	(0.0001)	(0.0238)	(0.0408)
Essential Worker × Lockdown	-0.0262	0.0014	-0.1672	-0.1706
	(0.0024)	(0.0004)	(0.0984)	(0.0690)
Observations	3028575	3060155	359535	2758846
Indiv. Chars	Yes	Yes	Yes	Yes
State FE	Yes	Yes	Yes	Yes
Region × Year FE	Yes	Yes	Yes	Yes
Month FE	Yes	Yes	Yes	Yes
Year FE	Yes	Yes	Yes	Yes
Interview Type FE	Yes	Yes	Yes	Yes
State COVID-19 Controls	Yes	Yes	Yes	Yes

Notes: Data from the Current Population Survey. Robust standard errors are in parentheses, adjusted for clustering by state. In column 1 the dependent variable is a dummy for whether the individual is unemployed. In column 2 the dependent variable is a dummy for whether the individual is in the labor force; were at work; held a job but were temporarily absent from work due to factors like vacation or illness; were seeking work; or were temporarily laid off from a job during the reference period. In the third column, the dependent variable is the hourly wages for individuals currently employed as wage/salary workers, paid hourly, and were in outgoing rotation groups. In the fourth column, the dependent variable is hours of work for individuals who are employed and either at work or absent from work during the survey week, all jobs. *Exposure* measures our exposure to disease index, *Proximity* is the proximity to coworkers index, *RemoteWork* is the remote work index, and *EssentialWorker* is our essential worker index, respectively. *Lockdown* is a dummy that is equal to one for the months after announcing a lockdown (measured by stay-at-home order announcement date). All columns include state, month, year, interview type and Census region × year fixed effects; the following demographic controls: gender, age, marital status, education and race; and the following state COVID-19 related controls: the number of COVID-19 tests performed per 10,000 inhabitants, implementation of mandatory face mask policies, social distancing measures, day care and school closures, and freezes on eviction and utilities. The time period is January 2016–June 2020.

We find that while the probability of being unemployed increases for everyone following a lockdown, those working more closely with others are more likely to be unemployed (4 percentage points) while those more able to work remotely, or are more likely to be an essential worker or are more exposed to disease/infection are less likely to be unemployed (2.3, 2.6, and 2.2 percentage points, respectively). For labor force participation, we find that those working more closely with others are less likely to be in the labor force while those more able to work remotely, those more likely to be essential workers and those more exposed to disease are more likely to participate in the labor market. These estimates are quite small economically however, representing changes of tenths of a percentage point in the probability of participation. We see changes in wages for essential workers and more exposed workers (lower) and workers in more proximity to others (higher) that are statistically significant at conventional levels but these likely represent compositional changes in the labor force. We do see a precisely estimated zero effect for remote workers. We also find lower hours worked for workers more likely to be essential but no statistically significant (at conventional levels) estimates for the other indexes. These estimates are also likely showing compositional changes in the work force rather than only direct effects of lockdowns on hours worked.

Table S5 in S1 Appendix presents the estimates for indexes included individually rather than all together. We observe a similar pattern of changes here as we did when comparing Table 9 and Table S3 in S1 Appendix–nearly identical estimates for probability of being unemployed and in the labor force participation for proximity, remote work, and essential work indexes, but changes for the exposure index. Again, we observe that without accounting for the other indexes, the estimated effect for the exposure indexes is not statistically significant for unemployment, labor force participation, or wages, though the estimate for hours worked remains statistically significant at the 10% level.

Turning to Table S6 in S1 Appendix, we find that the estimated effects of lockdowns are larger in absolute terms for healthcare based essential workers. However, these estimates are somewhat less precise than those for non-healthcare based essential workers and the estimates for non-healthcare based essential workers share signs with those for healthcare based workers. This suggests that while the effects of lockdowns are more pronounced for healthcare based essential workers, they are not solely responsible for the estimates, nor is the subgroup large enough to spur significant deviation from the estimates for non-healthcare based workers when combined.

### Discussion: Who is more affected and why?

Thus far, we have described the distribution and relationships between our indexes and major occupations and demographic characteristics in the Data and Identification Strategy section and presented estimates of how labor market outcomes changed after COVID-19’s arrival in the previous subsection. We now turn to the economic consequences of the pandemic across occupations and demographics, but more importantly present suggestive evidence that these indexes explain differences across these groups.

**Fig 11:**
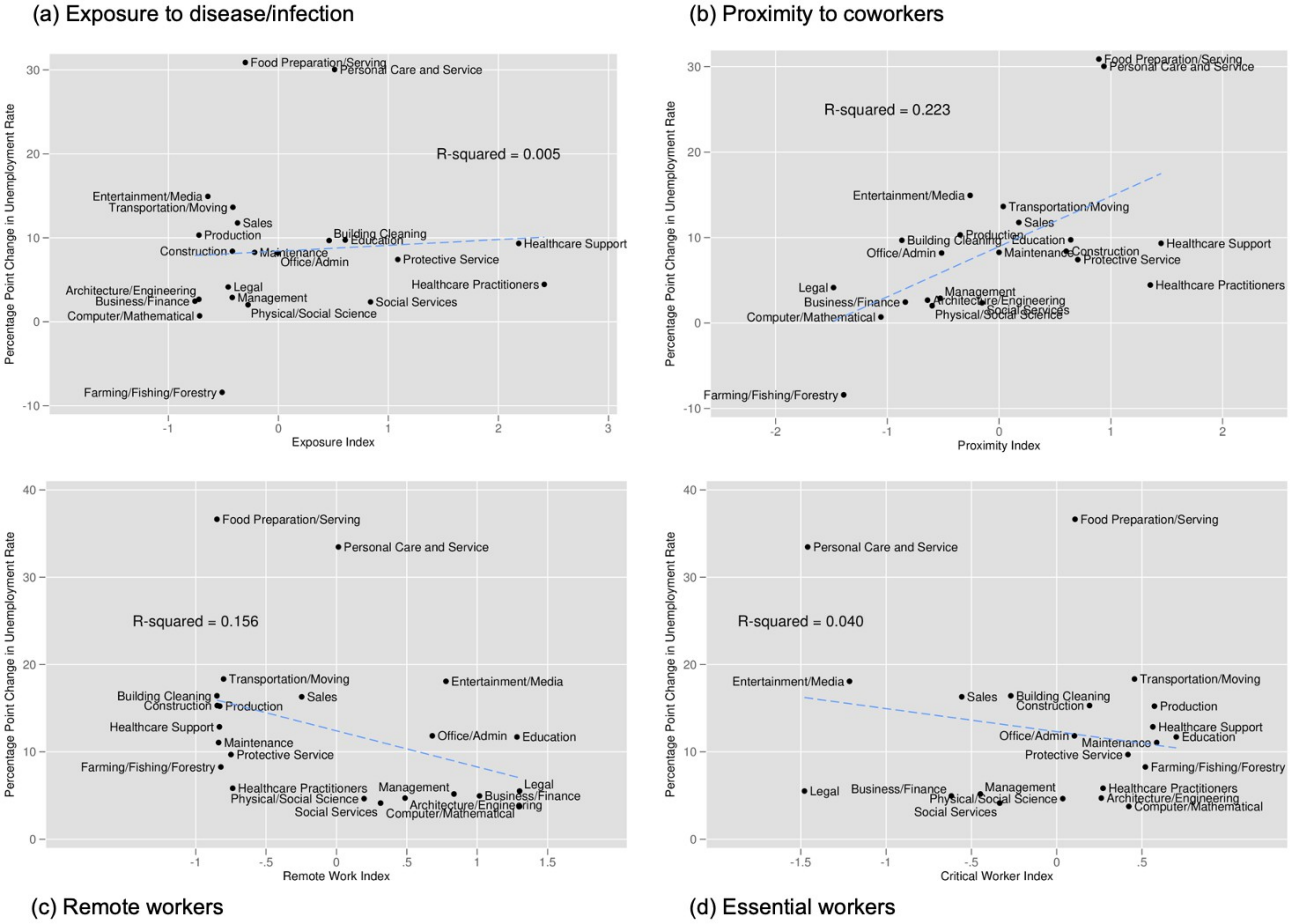
Percentage point change in unemployment (january 2020 to may 2020) by index and occupation. Notes: Unemployment data are from Current Population Survey, indexes are calculated by authors using data from O*NET, LMI, and [1]. The x-axis indicates the index value while the y-axis indicates the change in unemployment rate from January 2020 through May 2020. Each dot is a major occupation category (a two digit SOC code). Index values are normalized to mean 0 and standard deviation 1.

**Fig 12:**
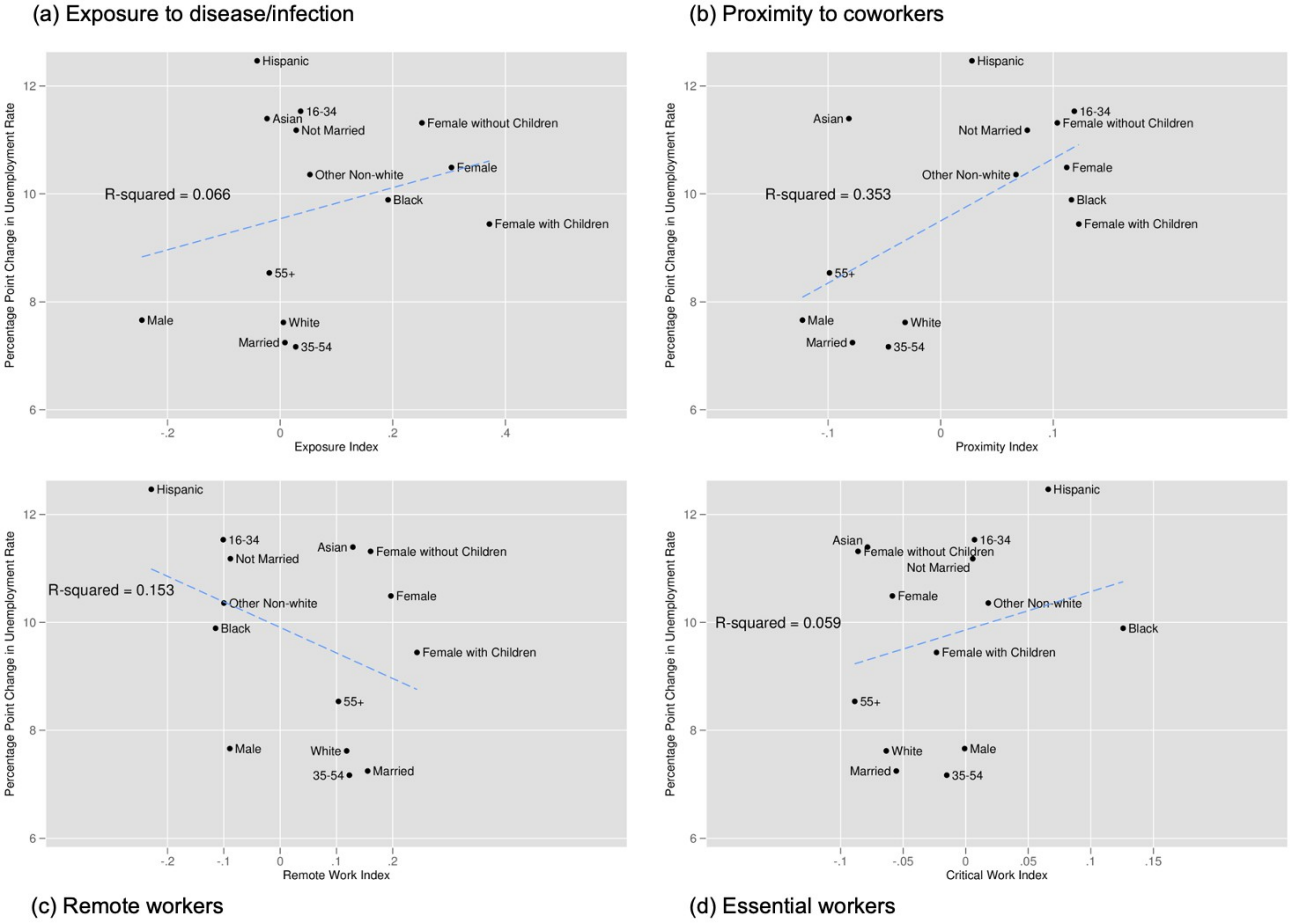
Percentage point change in unemployment (january 2020 to may 2020) by index and demographics. Notes: Unemployment data are from Current Population Survey, indexes are calculated by authors using data from O*NET, LMI, and [1]. The x-axis indicates the index value while the y-axis indicates the change in unemployment rate from January 2020 through May 2020. Each dot is a demographic characteristic. Index values are normalized to mean 0 and standard deviation 1.

Figs 11 and 12 plot the percentage point change in unemployment rates from January 2020 to May 2020 against our indexes across major occupations and demographic characteristics, respectively. These figures are similar to Fig 6 with the exception that the y-axis indicates the percentage point change in unemployment rates rather than the unemployment rate.

Looking first at Fig 11, we see that the ability to work remotely is strongly associated with smaller increases in unemployment during the pandemic (R^2^ of 0.156). Working more closely with others is associated with larger increases in unemployment rates across occupations and presents the strongest relationship among our indexes (R^2^ of 0.223). In contrast, our exposure to disease index is not strongly associated with the size of the change in unemployment across occupations. Occupations that are more essential are associated with smaller increases in unemployment but this relationship is not particularly strong (R^2^ of 0.040).

We now turn to Fig 12. Again, the ability to work remotely is tied to smaller increases in unemployment rates with some degree of strength (R^2^ of 0.1535). Exposure to disease is associated with larger increases in unemployment rates across demographic characteristics, though the strength of the relationship is not especially strong (R^2^ of 0.066). Working more closely to others is also positively related to changed in unemployment, and strongly so (R^2^ of 0.353). The weakest relationship between changes in unemployment and index values belongs to essential workers (R^2^ of 0.059) where we find that workers in more essential occupations are associated with larger increases in unemployment rates.

To sum up, and perhaps somewhat surprisingly, neither exposure to disease/infection nor essential workers indexes do a good job of explaining why some individuals or (major) occupations are affected by COVID-19. It is perhaps important to keep in mind the correlations between our indexes presented in the Data and Identification Strategy section, namely that our essential workers index is fairly strongly negatively associated with remote work while being positively (but less strongly) related to the exposure and proximity indexes. Of note, we did document that essential workers are less likely to have lost their job during the pandemic, suggesting that essential workers are overall less affected by the pandemic. Combining these previous results with those from Figs 11 and 12, this suggests that essential worker designation matters more within these major occupations and demographic characteristics rather than across them.

The ability to work remotely and physical proximity to others explain both the likelihood to lose one’s job and which people and occupations are most affected by the pandemic. These results provide plausible explanations for the heterogeneous effects of the pandemic on workers. Our indexes are explanations why certain demographic groups such as younger and minority workers have worse labor market outcomes during the pandemic (e.g., [3, 6, 8]). These demographic groups have lower ability to work remotely, as shown in Fig 12.

## Conclusion

In this paper, we explore the economic consequences of COVID-19 and lockdowns on employment and wages in the United States. We investigate differential effects for specific occupations or occupational tasks, using four indexes: workers able to work remotely ([1]); workers relatively more exposed to disease, workers that work with proximity to coworkers and essential/critical workers.

Our analysis documents heterogeneous effects of COVID-19 across occupations and workers. In particular, we find that individuals able to work remotely and essential workers are less affected by the pandemic. We also highlight a key occupational task factor explaining larger labor market effects: being in an occupation working in proximity to others. Additionally, we document the effect of the pandemic on labor market outcomes of critical workers in health sector versus non health sector and find lower unemployment decrease in the health care sector. Furthermore, we find that the labor market effects of the pandemic are larger in areas with larger number of cases and death.

These results could lead workers to change (and students to choose different) occupation in the short- or medium-term, and move into less ‘risky’ ones. Similarly, COVID-19 may accelerate the rise in flexible work arrangements and telecommuting ([31, 32]). Future work needs to investigate if the effect will lead to permanent or temporary shift in the labor market. The pandemic might also discourage some workers to enter the health care profession, as the working conditions have deteriorated during the pandemic (e.g., [33]).

## Supporting information

S1 Appendix (PDF)
