# Supplementary material for: The short-term economic consequences of COVID-19: Exposure to disease, remote work and government response
Source: PLoS One. 2023 Mar 15;18(3):e0270341. doi: 10.1371/journal.pone.0270341 (PMC10016649; doi:10.1371/journal.pone.0270341)
Supplement: S1 Appendix — (PDF) [file pone.0270341.s001.pdf]

S1 Appendix

O*NET Index calculations

O*NET's indexes for "Exposure to infection and disease" and "Physical proximity to others" are created based on survey responses. These survey responses are collected on a 1–5 scale (the values for each questions are shown in Figures S1 and S2) and then converted into an index with the following formula:

$S = ((O - 1)/(H - 1)) * 100$

where S is the new index value, O is the original score on the 1–5 scale, and H is the highest possible score. As an example, a collected score of 4 becomes 75 $(= (4 - 1)/(5 - 1)) * 100)$.

We note that while we do not aggregate any of our indexes, some respondents give insufficient detail to have their occupation placed in the most detailed occupational code and are placed in a broader category. These groups are given weighted values of our indexes as described in the text.

COVID-19 and Work Arrangement

Via email correspondence, the BLS indicated that "BLS (working with the Census Bureau) supplied special instructions to CPS interviewers regarding questions related to the measurement of persons at work part time for economic reasons and persons on temporary layoff. Those instructions are described here:

https://www.bls.gov/cps/employment-situation-covid19-faq-march-2020.pdf (Question 7).

We did not supply special Coronavirus-related guidance regarding other labor force questions in the survey.

The basic CPS does not regularly include questions regarding telework/work at home. However, there is a proposal to add questions to the survey beginning with the collection of data for May; if approved, these questions should provide some insight into telework and other labor market developments associated with the pandemic:

https://www.reginfo.gov/public/do/PRAViewDocument?ref_nbr=202004-1220-008 (see supporting statement A).

Appendix Figure S6 presents the share of workers whose usual work activities or duties have changed since last month. Typically, just under 1% of respondents are changing their work activities. However, since December 2019 this has doubled, from 0.7% to nearly 1.4%, with a 0.2 percentage point increase in March 2020.

Appendix Table S7 presents the results for a dependent variable that equals one if an individual has changed work activities or duties since the previous month. The top panel shows an increase in the probability of workers reporting changes in work activities after COVID-19 (an increase of about 0.3 percentage points, statistically significant at the 5% level). For *Known cases per* 10,000 *lagged* 2 *weeks*, we're seeing positive point estimates that are neither economically meaningful nor statistically significant at conventional levels.

Appendix Table S8 provides estimates for the interaction of our indexes with *Post COVID*. None of the interactions are statistically significant below the 50% level and they are all at least an order of magnitude smaller than the *Post COVID*

effect. In terms of signs, all interactions are positive but the exposure control is negative while the essential worker control is positive. This indicates that workers who are more exposed are less likely to see work arrangement changes while those who are essential workers are more likely to see see their arrangements change. However, we are not able to detect a difference workers along our indexes before and after COVID-19.

Employment and Wages: State-Level Cases and Deaths

We also investigate the impact of COVID-19 at the state-level. In this analysis, we exploit variation in cases and fatalities over time across states, which we lag by 2 weeks to account for any delay in labor market adjustment to perceived COVID-19 conditions. The model is:

$$Y_{i,s,t} = \alpha + \beta CASES_{s,t} + X'_{i,s,t}\gamma + \theta_s + \delta_t + \varepsilon_{i,s,t}, \quad (3)$$

where $y_{i,s,t}$ is an economic outcome for individual i in state s and month t. *Lagged* $CASES_{s,t}$ is the number of confirmed cases per 10,000 inhabitants lagged 2 weeks in state s in time t. In some model, we replace the variable $CASES_{s,t}$ by $DEATHS_{s,t}$, which capture the number of lagged COVID-19 fatalities per 10,000 inhabitants. Finally, θ_s and δ_t represent state and time fixed effects, respectively.

Appendix Figure S7 plots our labor market outcomes for individuals split by states with cumulative known COVID-19 case rates above and below the median. We find that states above the median case rate experienced a larger increase in unemployment and a larger decrease in labor force participation. Looking more specifically at the change from February 2020 to April 2020, states with above median case rates saw an 11 percentage point increase in unemployment against 8.5 percentage points for states below the median. The difference in hours worked per week is also more pronounced for the states above the median.

We confirm these results in Table S9, following equation (3). The variables of interest are the number of cumulative COVID-19 cases (columns 1, 2, 5 and 6) and deaths (columns 3, 4, 7 and 8) per 10,000 inhabitants. We also include the number of cumulative COVID-19 cases and deaths per 10,000 inhabitants squared, both lagged by 2 weeks. We find a positive coefficient on the squared term and a negative coefficient on the coefficient for unemployment, suggesting that layoffs are speeding up for each new case or death. We find that the cumulative number of lagged cases and deaths at the state-level is positively associated to the unemployment rate and positively related to labor force participation and hours of work, suggesting that individuals in states with more COVID-19 cases were more likely to lose their jobs but were not leaving the labor force.

Impacts by Occupation: State-Level cases

Appendix Tables S10 - S13 provide estimates for the differential effects of COVID-19 on workers across our exposure, proximity, essential workers and remote work indexes using the cumulative known COVID-19 cases or deaths per 10,000 inhabitants (lagged 2 weeks), instead of *Post COVID*. We find that occupations that work in proximity to others are more likely to be unemployed due to COVID-19. We also find that workers in occupations that can work remotely

Figure S1: O*NET Survey Question Used for Exposure to Disease

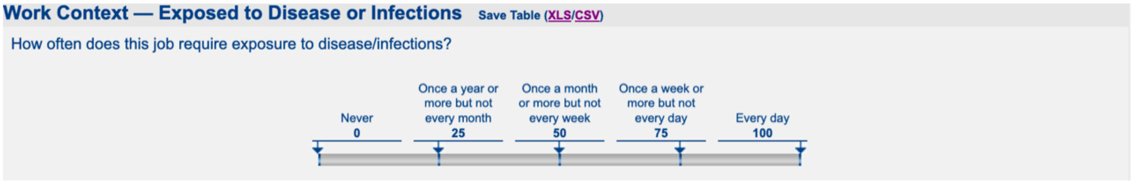

Notes: Survey question asking "How often does this job require exposure to disease/infections?" with five possible answers: (1) Never, (2) Once a year or more but not every month, (3) Once a month or more but not every week, (4) Once a week or more but not every day, and (5) Every day.

are less likely to be unemployed and work more hours. The effects are significant at the 1% level. These results are in line with our national-level analysis. We also find that in states with larger deaths per capita, essential workers are less likely to be unemployed.

Appendix Tables S14 and S15 provide estimates analogous to those of Table 9, including all indexes and interactions in the same regression equation. Looking at Appendix Table S14, we see exactly the same patterns as individual regressions for labor force participation, wages, and hours worked. For unemployment, the exposure index interaction is now negative and statistically significant, the same style of change we saw previously in Table 9. The inferences for the other indexes remain the same for unemployment.

Switching to Appendix Table S15 we find again that same pattern of unchanging inference for all indexes except the exposure index, which is now negative and statistically significant for unemployment. For labor force participation, the signs of the estimates remain the same and the estimates are similar in magnitude, though the inclusion of the other indexes has reduced the standard errors and these results are now statistically significant (except remote work).

Lastly, Appendix Tables S16 and S17 provide breakdowns of the estimates for the essential worker index by healthcare and non-healthcare based workers. Healthcare based essential workers deemed more essential are less likely to be unemployed in areas with higher case rates than those deemed less essential. This likelihood is also lower than that of more essential non-healthcare based workers over less essential non-healthcare workers. This is also true of areas with larger death rates. The sign of estimates for the other labor market outcomes are also the same for healthcare based and non-healthcare based essential workers, though the magnitudes for labor force participation and wages are larger in absolute values for healthcare based workers (true of interactions with case and death rates) while the reverse is true for hours worked. Combining this information, it appears that our results are more pronounced for healthcare based workers but the effects are still present (though less pronounced) for non-healthcare based essential workers.

Figure S2: O*NET Survey Question Used for Physical Proximity

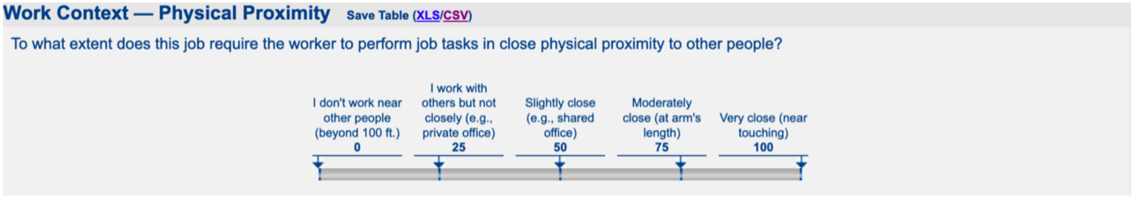

Notes: Survey question asking “How physically close to other people are you when you perform your current job?” with five possible responses: (1) I don’t work near other people(beyond 100 ft.), (2) I work with others but not closely (e.g., private office), (3) Slightly close (e.g., shared office), (4) Moderately close(at arm’s length), and (5) Very close (near touching).

Figure S3: Physical Proximity, Exposure to the Disease and Essential Workers by Occupation

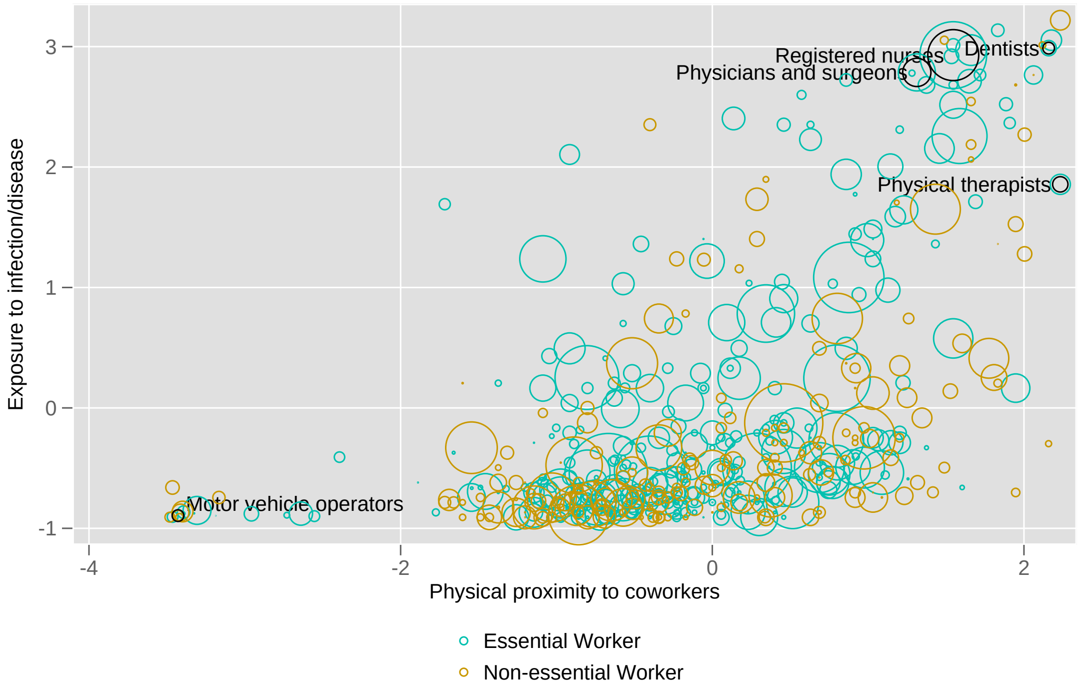

Notes: Each circle represents an occupation. The size of each circle represents the number of CPS respondents employed in that occupation–the larger the circle, the greater the number of people employed in that occupation. The x-axis plots each occupation’s physical proximity to coworkers, measured by O*NET’s index. The further to the right, the closer in proximity employees in that occupation work with their coworkers. The y-axis plots each occupation’s exposure to infection and disease, also measured by O*NET’s index. The further up, the more frequently employees in that occupation are exposes to infection and disease. The color of the circles corresponds to whether or not the occupation is considered essential by the Labor Market Information Institute.

Figure S4: Remote Work, Exposure to the Disease and Essential Workers by Occupation

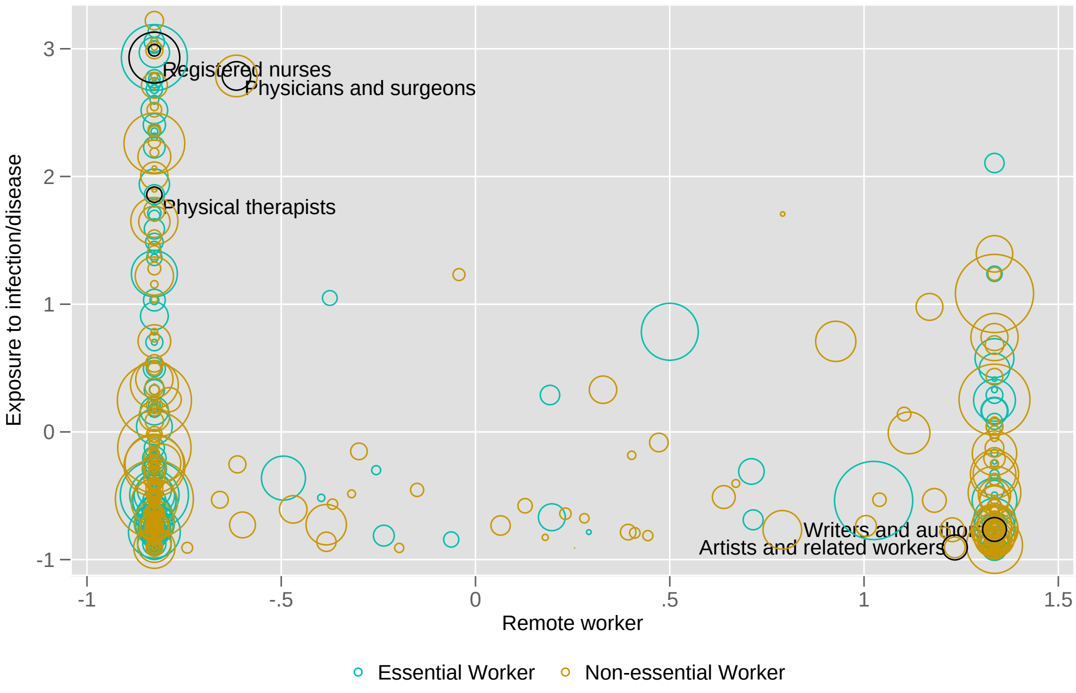

Notes: Each circle represents an occupation. The size of each circle represents the number of CPS respondents employed in that occupation–the larger the circle, the greater the number of people employed in that occupation. The x-axis plots each occupation's ability to work remotely. The further to the right, the more easily that occupation can be done remotely. The y-axis plots each occupation's exposure to infection and disease, also measured by O*NET's index. The further up, the more frequently employees in that occupation are exposes to infection and disease. The color of the circles corresponds to whether or not the occupation is considered essential by the Labor Market Information Institute.

Figure S5: Real Hourly Wages by Index and Occupation

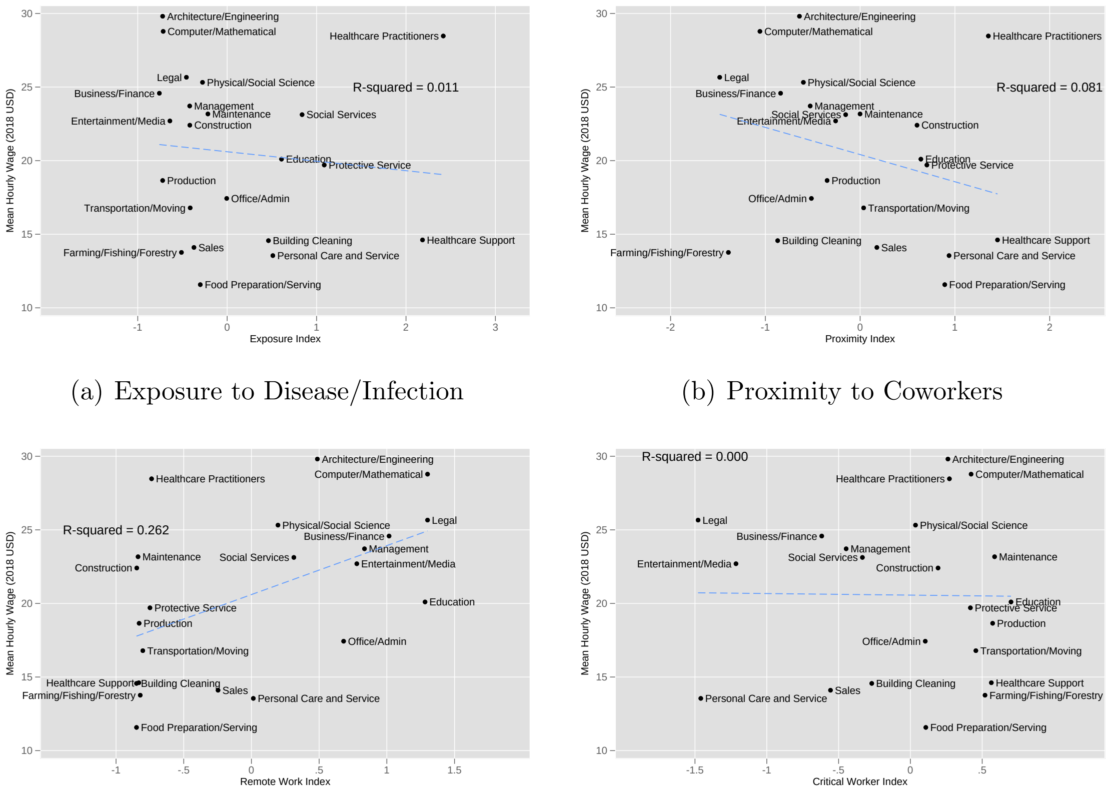

(a) Exposure to Disease/Infection

(b) Proximity to Coworkers

(c) Remote Workers

(d) Essential Workers

Notes: Wage data are from Current Population Survey, indexes are calculated by authors using data from O*NET, LMI, and **?**. The x-axis indicates the index value while the y-axis indicates the average real hourly wage rate (in 2018 dollars) from March 2020 through June 2020. Each dot is a major occupation category (a two digit SOC code). Index values are normalized to mean 0 and standard deviation 1.

Figure S6: COVID-19 and Work Arrangement

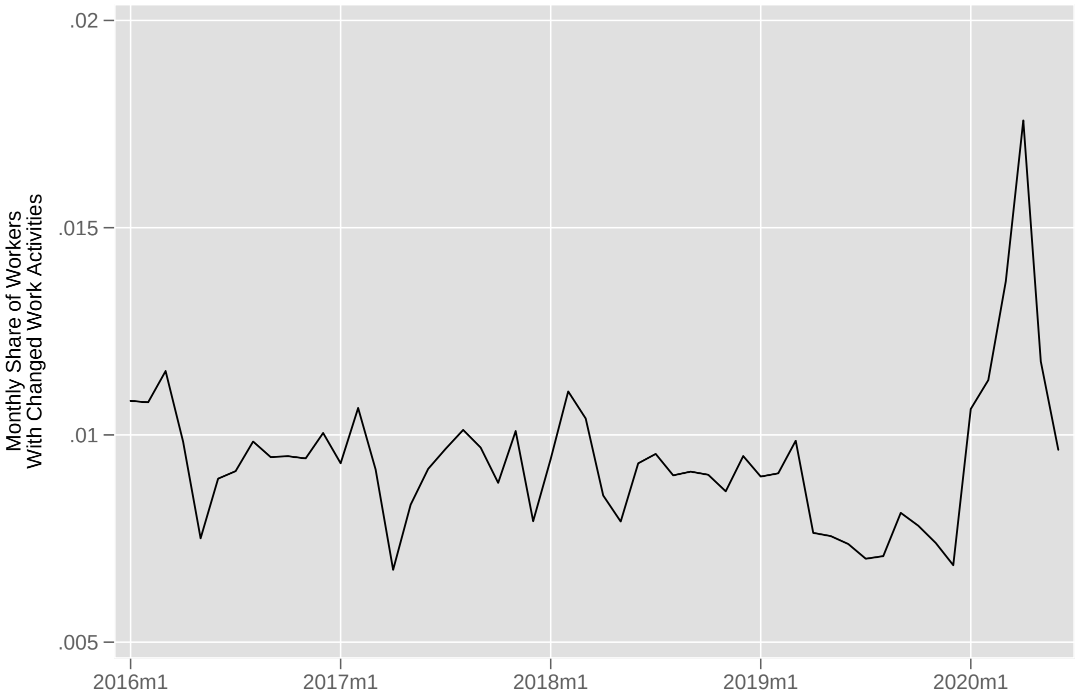

Notes: The y-axis plots the share of respondents who answered ”Yes” to the question ”Have the usual activities and duties of your job changed since last month?”

Figure S7: Unemployment Rate, Labor Force Participation, Hours of Work and Hourly Wages by State COVID-19 Case Rate.

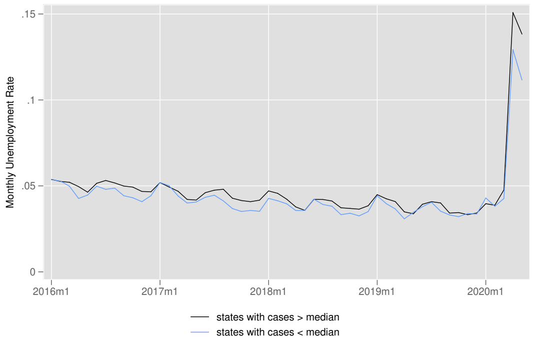

(a) Unemployment Rate.

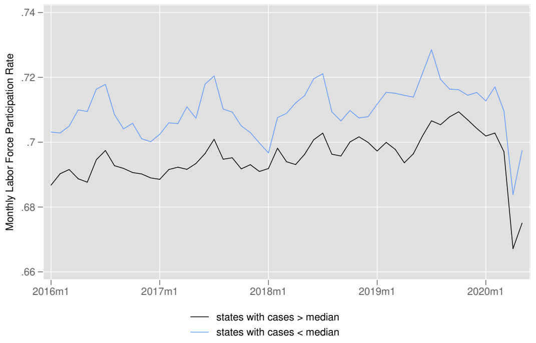

(b) Labor Force Participation.

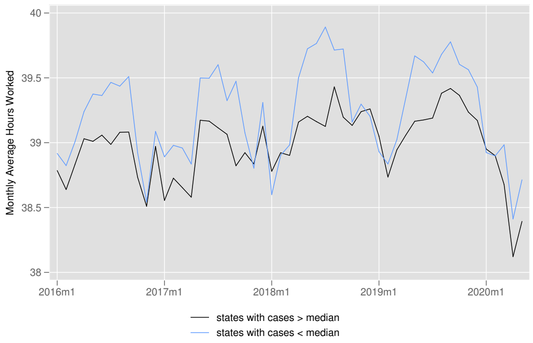

(c) Hours of Work.

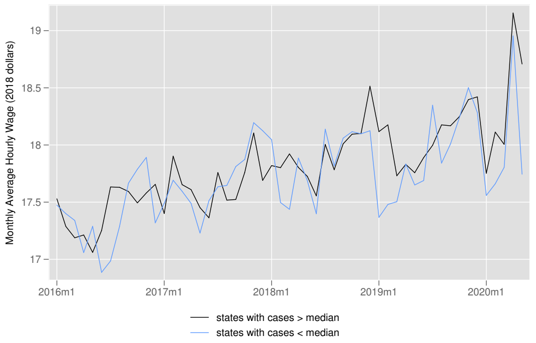

(d) Hourly Wages.

Notes: Authors' calculations. Data from the Current Population Survey. The time period is January 2016 to June 2020. Panel A plots the unemployment rate in states above and below the April 2020 median for cumulative number of known COVID-19 cases per 10,000 inhabitants. Panel B plots the labor force participation in states above and below the April 2020 median for cumulative number of known COVID-19 cases per 10,000 inhabitants. Individuals in the labor force were at work; held a job but were temporarily absent from work due to factors like vacation or illness; were seeking work; or were temporarily laid off from a job during the reference period. Panel C plots hours work in states above and below the April 2020 median for cumulative number of known COVID-19 cases per 10,000 inhabitants. Hours work: civilians aged 16–70 who are employed and either at work or absent from work during the survey week, all jobs. Trimmed to exclude values below 1st percentile and above 99th percentile. Panel D plots hourly wages in states above and below the April 2020 median for cumulative number of known COVID-19 cases per 10,000 inhabitants. Hourly wages: civilians aged 16–70 currently employed as wage/salary workers, paid hourly, and were in outgoing rotation groups. Excludes self-employed persons. Trimmed to exclude values below 1st percentile and above 99th percentile. Reported in 2018 constant dollars.

Table S1: Index for Exposure to Disease 60th to 40th Percentiles

**Occupation**	**Score**
Directors, religious activities and education	13
Telephone operators	12
Locomotive engineers and operators	11.5
Chemical processing machine setters, operators, and tenders	11.2
Chemical engineers	11
Conservation scientists and foresters	10.2
Other teachers and instructors	10.1
First-line supervisors of retail sales workers	10
News analysts, reporters and correspondents	9.1
Athletes, coaches, umpires, and related workers	9.0
Bus and truck mechanics and diesel engine specialists	9
Baggage porters, bellhops, and concierges	8.5
Announcers	8.3
Chemical technicians	8
Small engine mechanics	7.1
Fundraisers	7
Drywall installers, ceiling tile installers, and tapers	6.4
Sales and related workers, all other	6.3
Plasterers and stucco masons	6
Pumping station operators	5.7

Notes: This index is taken from a survey question asking "How physically close to other people are you when you perform your current job?" with five possible responses: (1) I don't work near other people (beyond 100 ft.), (2) I work with others but not closely(e.g., private office), (3) Slightly close (e.g., shared office), (4) Moderately close (at arm's length), and (5) Very close (near touching). One example occupation is shown for each percentile for space considerations.

Table S2: Index for Physical Proximity 60th to 40th Percentiles

**Occupation**	**Score**
Radio and telecommunications equipment installers and repairers	62.4
Library technicians	62
Computer support specialists	61.9
Plasterers and stucco masons	61
Medical, dental, and ophthalmic laboratory technicians	60
Stationary engineers and boiler operators	60
Billing and posting clerks	59
Court, municipal, and license clerks	58.6
Insulation workers	58.4
Wholesale and retail buyers, except farm products	58
Television, video, and motion picture camera operators and editors	57.1
Power plant operators, distributors, and dispatchers	57.0
Financial examiners	57
Agricultural and food science technicians	56.5
Prepress technicians and workers	56
Molders, shapers, and casters, except metal and plastic	55.7
Farmers, ranchers, and other agricultural managers	55.6
Lifeguards and other recreational, and all other protective service workers	55.6
Shoe and leather workers and repairers	55
Jewelers and precious stone and metal workers	54.3
Computer systems analysts	54

Notes: This index is taken from a survey question asking "How physically close to other people are you when you perform your current job?" with five possible responses: (1) I don't work near other people (beyond 100 ft.), (2) I work with others but not closely(e.g., private office), (3) Slightly close (e.g., shared office), (4) Moderately close (at arm's length), and (5) Very close (near touching). One example occupation is shown for each percentile for space considerations.

Table S3: The Impacts of COVID-19: Exposure, Proximity, Remote Work and Essential Workers

Panel A. Unemployed	Baseline	Exposure	Proximity	Remote Work	Essential Worker
Post COVID	0.0781	0.0779	0.0780	0.0795	0.0784
	(0.0038)	(0.0038)	(0.0038)	(0.0040)	(0.0038)
Index		-0.0037	0.0025	-0.0080	0.0007
		(0.0002)	(0.0004)	(0.0003)	(0.0003)
Index × Post		0.0007	0.0225	-0.0216	-0.0136
		(0.0009)	(0.0018)	(0.0019)	(0.0017)
Observations	3158372	3146035	3146035	3029863	3028575

Panel B. Labor Force Participation	Baseline	Exposure	Proximity	Remote Work	Essential Worker
Post COVID	-0.0311	-0.0052	-0.0052	-0.0053	-0.0052
	(0.0034)	(0.0006)	(0.0006)	(0.0006)	(0.0006)
Index		0.0002	-0.0010	0.0014	0.0003
		(0.0001)	(0.0001)	(0.0001)	(0.0001)
Index × Post		-0.0002	-0.0018	0.0019	0.0004
		(0.0003)	(0.0004)	(0.0004)	(0.0003)
Observations	4506713	3179089	3179089	3061498	3060155

Panel C. Wages	Baseline	Exposure	Proximity	Remote Work	Essential Worker
Post COVID	0.4739	0.4750	0.4413	0.4966	0.4989
	(0.0871)	(0.0895)	(0.0895)	(0.0932)	(0.1011)
Index		1.0141	-0.2231	1.3559	0.4012
		(0.0349)	(0.0345)	(0.0963)	(0.0210)
Index × Post		-0.0515	0.1502	0.0450	-0.1818
		(0.0928)	(0.0717)	(0.0903)	(0.0967)
Observations	374408	374408	374408	359578	359535

Panel D. Hours	Baseline	Exposure	Proximity	Remote Work	Essential Worker
Post COVID	-0.8761	-0.8750	-0.9255	-0.9298	-0.8991
	(0.0845)	(0.0841)	(0.0828)	(0.0903)	(0.0964)
Index		-0.3008	-0.9866	0.9968	0.2720
		(0.0347)	(0.0520)	(0.0350)	(0.0463)
Index × Post		-0.0710	-0.0401	0.0509	-0.3579
		(0.0598)	(0.0690)	(0.0677)	(0.0598)
Observations	2865884	2865884	2865884	2759760	2758846
Indiv. Chars	Yes	Yes	Yes	Yes	Yes
State FE	Yes	Yes	Yes	Yes	Yes
Region × Year FE	Yes	Yes	Yes	Yes	Yes
Month FE	Yes	Yes	Yes	Yes	Yes
Year FE	Yes	Yes	Yes	Yes	Yes
Interview Type FE	Yes	Yes	Yes	Yes	Yes
State COVID-19 Controls	Yes	Yes	Yes	Yes	Yes

Notes: Data from the Current Population Survey. Robust standard errors are in parentheses, adjusted for clustering by state. In the top panel, the dependent variable is a dummy for whether the individual is unemployed. In the second, the dependent variable is a dummy for whether the individual is in the labor force; were at work; held a job but were temporarily absent from work due to factors like vacation or illness; were seeking work; or were temporarily laid off from a job during the reference period. In the third panel, the dependent variable is the hourly wages for individuals currently employed as wage/salary workers, paid hourly, and were in outgoing rotation groups. In the bottom panel, the dependent variable is hours of work for individuals who are employed and either at work or absent from work during the survey week, all jobs. In column 1 of all panels, we provide baseline estimates without the indexes. Columns 2–5 provide estimates for our indexes. *Index* measures our exposure to disease index, proximity to coworkers index, remote work index, and essential worker index, respectively. *Post COVID* is a dummy that is equal to one for the months after March 2020. All columns include state, month, year, interview type and Census region × year fixed effects and the following demographic controls: gender, age, marital status, education and race; and the following state COVID-19 related controls: the number of COVID-19 tests performed per 10,000 inhabitants, implementation of mandatory face mask policies, social distancing measures, day care and school closures, and freezes on eviction and utilities. The time period is January 2016–June 2020.

Table S5: The Impacts of Lockdowns: Exposure, Proximity, Remote and Essential Work

	Baseline	Exposure	Proximity	Remote Work	Essential Worker
Panel A. Unemployed					
Lockdown	0.0683	0.0686	0.0691	0.0716	0.0694
	(0.0165)	(0.0164)	(0.0165)	(0.0171)	(0.0167)
Index		-0.0035	0.0006	-0.0035	-0.0007
		(0.0003)	(0.0004)	(0.0003)	(0.0003)
Index × Lockdown		0.0013	0.0304	-0.0294	-0.0178
		(0.0013)	(0.0025)	(0.0025)	(0.0023)
Observations	3158372	3146035	3146035	3029863	3028575
Panel B. Labor Force Participation	Baseline	Exposure	Proximity	Remote Work	Essential Worker
Lockdown	-0.0235	-0.0080	-0.0080	-0.0083	-0.0082
	(0.0078)	(0.0017)	(0.0016)	(0.0017)	(0.0017)
Index		-0.0001	-0.0004	0.0001	0.0003
		(0.0000)	(0.0001)	(0.0001)	(0.0001)
Index × Lockdown		0.0001	-0.0024	0.0025	0.0007
		(0.0003)	(0.0004)	(0.0005)	(0.0004)
Observations	4506713	3179089	3179089	3061498	3060155
Panel C. Wages	Baseline	Exposure	Proximity	Remote Work	Essential Worker
Lockdown	0.3910	0.3809	0.3751	0.3461	0.4102
	(0.2300)	(0.2265)	(0.2278)	(0.2146)	(0.2288)
Index		0.6404	-0.0631	0.6305	0.4954
		(0.0359)	(0.0368)	(0.0704)	(0.0208)
Index × Lockdown		-0.1124	0.1249	-0.0091	-0.2139
		(0.0832)	(0.0633)	(0.1490)	(0.0879)
Observations	374408	374408	374408	359578	359535
Panel D. Hours	Baseline	Exposure	Proximity	Remote Work	Essential Worker
Lockdown	-0.9072	-0.9180	-0.9623	-0.9547	-0.9313
	(0.2172)	(0.2176)	(0.2221)	(0.2269)	(0.2272)
Index		-0.3896	-0.6943	0.4259	0.3341
		(0.0302)	(0.0530)	(0.0316)	(0.0408)
Index × Lockdown		-0.1140	-0.1107	0.0655	-0.3012
		(0.0667)	(0.0780)	(0.0758)	(0.0688)
Observations	2865884	2865884	2865884	2759760	2758846
Indiv. Chars	Yes	Yes	Yes	Yes	Yes
State FE	Yes	Yes	Yes	Yes	Yes
Region × Year FE	Yes	Yes	Yes	Yes	Yes
Month FE	Yes	Yes	Yes	Yes	Yes
Year FE	Yes	Yes	Yes	Yes	Yes
Interview Type FE	Yes	Yes	Yes	Yes	Yes
State COVID-19 Controls	Yes	Yes	Yes	Yes	Yes

Notes: Data from the Current Population Survey. Robust standard errors are in parentheses, adjusted for clustering by state. In the top panel, the dependent variable is a dummy for whether the individual is unemployed. In the second, the dependent variable is a dummy for whether the individual is in the labor force; were at work; held a job but were temporarily absent from work due to factors like vacation or illness; were seeking work; or were temporarily laid off from a job during the reference period. In the third panel, the dependent variable is the hourly wages for individuals currently employed as wage/salary workers, paid hourly, and were in outgoing rotation groups. In the bottom panel, the dependent variable is hours of work for individuals who are employed and either at work or absent from work during the survey week, all jobs. In column 1 of all panels, we provide baseline estimates without the indexes. Columns 2–5 provide estimates for our indexes. *Index* measures our exposure to disease index, proximity to coworkers index, remote work index, and essential worker index, respectively. *Lockdown* is a dummy that is equal to one for the months after announcing a lockdown (measured by stay-at-home order announcement date). All columns include state, month, year, interview type and Census region × year fixed effects; the following demographic controls: gender, age, marital status, education and race; and the following state COVID-19 related controls: the number of COVID-19 tests performed per 10,000 inhabitants, implementation of mandatory face mask policies, social distancing measures, day care and school closures, and freezes on eviction and utilities. The time period is January 2016–June 2020.

Table S4: The Impacts of COVID-19: Healthcare and Non-Healthcare Essential Workers

Panel A. Unemployed	Essential Worker	Healthcare Essential Worker	Non-Healthcare Essential Worker
Post COVID	0.0259	0.0271	0.0255
	(0.0041)	(0.0042)	(0.0040)
Index	-0.0005	-0.0073	-0.0003
	(0.0003)	(0.0009)	(0.0003)
Index × Post	-0.0141	-0.0338	-0.0127
	(0.0017)	(0.0047)	(0.0016)
Observations	3028575	3154563	3032384
Panel B. Labor Force Participation	Essential Worker	Healthcare Essential Worker	Non-Healthcare Essential Worker
Post COVID	-0.0020	-0.0156	-0.0020
	(0.0008)	(0.0024)	(0.0008)
Index	0.0003	0.1575	0.0003
	(0.0001)	(0.0056)	(0.0001)
Index × Post	0.0005	-0.0010	0.0004
	(0.0003)	(0.0053)	(0.0003)
Observations	3060155	4502853	3064015
Panel C. Wages	Essential Worker	Healthcare Essential Worker	Non-Healthcare Essential Worker
Post COVID	0.0668	0.1001	0.0570
	(0.2034)	(0.1990)	(0.2017)
Index	0.4973	2.5373	0.3603
	(0.0214)	(0.1332)	(0.0244)
Index × Post	-0.1706	-1.0072	-0.1424
	(0.0694)	(0.3500)	(0.0668)
Observations	359535	373639	360304
Panel D. Hours	Essential Worker	Healthcare Essential Worker	Non-Healthcare Essential Worker
Post COVID	-0.5148	-0.4880	-0.5229
	(0.1067)	(0.0962)	(0.1045)
Index	0.3380	-0.5014	0.3882
	(0.0405)	(0.1152)	(0.0447)
Index × Post	-0.2629	-0.2334	-0.2538
	(0.0585)	(0.1959)	(0.0632)
Observations	2758846	2862358	2762372
Indiv. Chars	Yes	Yes	Yes
State FE	Yes	Yes	Yes
Interview Type FE	Yes	Yes	Yes
Region × Year FE	Yes	Yes	Yes
Month FE	Yes	Yes	Yes
Year FE	Yes	Yes	Yes
State COVID-19 Controls	Yes	Yes	Yes

Notes: Data from the Current Population Survey. Robust standard errors are in parentheses, adjusted for clustering by state. In the top panel, the dependent variable is a dummy for whether the individual is unemployed. In the second, the dependent variable is a dummy for whether the individual is in the labor force; were at work; held a job but were temporarily absent from work due to factors like vacation or illness; were seeking work; or were temporarily laid off from a job during the reference period. In the third panel, the dependent variable is the hourly wages for individuals currently employed as wage/salary workers, paid hourly, and were in outgoing rotation groups. In the bottom panel, the dependent variable is hours of work for individuals who are employed and either at work or absent from work during the survey week, all jobs. *Index* measures is our essential worker index for the version denoted in the column titles. Column 1 contains estimates for all essential workers, column 2 for essential workers in healthcare, and column 3 for essential workers not in healthcare. *Post COVID* is a dummy that is equal to one for the months after March 2020. All columns include state, month, year, interview type and Census region × year fixed effects; the following demographic controls: gender, age, marital status, education and race; and the following state COVID-19 related controls: the number of COVID-19 tests performed per 10,000 inhabitants, implementation of mandatory face mask policies, social distancing measures, day care and school closures, and freezes on eviction and utilities. The time period is January 2016–June 2020.

Table S6: The Impacts of Lockdowns: Healthcare and Non-Healthcare Essential Workers

Panel A. Unemployed

	Essential Worker	Healthcare Essential Worker	Non-Healthcare Essential Worker
Index	-0.0007	-0.0076	-0.0004
	(0.0003)	(0.0009)	(0.0003)
Lockdown	0.0694	0.0698	0.0688
	(0.0167)	(0.0165)	(0.0167)
Index × Lockdown	-0.0178	-0.0432	-0.0160
	(0.0023)	(0.0065)	(0.0022)
Observations	3028575	3154563	3032384

Panel B. Labor Force Participation

	Essential Worker	Healthcare Essential Worker	Non-Healthcare Essential Worker
Index	0.0003	0.1572	0.0003
	(0.0001)	(0.0055)	(0.0001)
Lockdown	-0.0082	-0.0237	-0.0081
	(0.0017)	(0.0078)	(0.0018)
Index × Lockdown	0.0007	0.0028	0.0005
	(0.0004)	(0.0054)	(0.0005)
Observations	3060155	4502853	3064015

Panel C. Wages

	Essential Worker	Healthcare Essential Worker	Non-Healthcare Essential Worker
Index	0.4954	2.5213	0.3587
	(0.0208)	(0.1323)	(0.0239)
Lockdown	0.4102	0.4352	0.4122
	(0.2288)	(0.2291)	(0.2286)
Index × Lockdown	-0.2139	-1.1921	-0.1739
	(0.0879)	(0.3930)	(0.0849)
Observations	359535	373639	360304

Panel D. Hours

	Essential Worker	Healthcare Essential Worker	Non-Healthcare Essential Worker
Index	0.3341	-0.4986	0.3842
	(0.0408)	(0.1135)	(0.0448)
Lockdown	-0.9313	-0.8767	-0.9552
	(0.2272)	(0.2189)	(0.2267)
Index × Lockdown	-0.3012	-0.3966	-0.2840
	(0.0688)	(0.2268)	(0.0742)
Observations	2758846	2862358	2762372
Indiv. Chars	Yes	Yes	Yes
State FE	Yes	Yes	Yes
Interview Type FE	Yes	Yes	Yes
State COVID-19 Controls	Yes	Yes	Yes
Region × Year FE	Yes	Yes	Yes
Month FE	Yes	Yes	Yes
Year FE	Yes	Yes	Yes

Notes: Data from the Current Population Survey. Robust standard errors are in parentheses, adjusted for clustering by state. In the top panel, the dependent variable is a dummy for whether the individual is unemployed. In the second, the dependent variable is a dummy for whether the individual is in the labor force; were at work; held a job but were temporarily absent from work due to factors like vacation or illness; were seeking work; or were temporarily laid off from a job during the reference period. In the third panel, the dependent variable is the hourly wages for individuals currently employed as wage/salary workers, paid hourly, and were in outgoing rotation groups. In the bottom panel, the dependent variable is hours of work for individuals who are employed and either at work or absent from work during the survey week, all jobs. *Index* measures is our essential worker index for the version denoted in the column titles. Column 1 contains estimates for all essential workers, column 2 for essential workers in healthcare, and column 3 for essential workers not in healthcare. *Lockdown* is a dummy that is equal to one for the months after announcing a lockdown (measured by stay-at-home order announcement date). All columns include state, month, year, interview type and Census region × year fixed effects; the following demographic [illegible]

Table S7: COVID-19 and Work Arrangement: National-Level

	Usual Work Changed Since Last Month		
	(1)	(2)	(3)
Post COVID	0.00348	0.00410	0.00411
	(0.00069)	(0.00067)	(0.00067)
Observations	1845499	1845499	1845499
	(1)	(2)	(3)
Known cases per 10,000 lagged 2 weeks	0.00002	0.00002	0.00001
	(0.00001)	(0.00001)	(0.00001)
Observations	1845499	1845499	1845499
Indiv. Chars	No	Yes	Yes
State FE	Yes	Yes	Yes
Region × Year FE	No	No	Yes
Month FE	Yes	Yes	Yes
Year FE	Yes	Yes	Yes
Interview Type FE	No	Yes	Yes
State COVID-19 Controls	No	No	Yes

Notes: Data from the Current Population Survey. Robust standard errors are in parentheses, adjusted for clustering by state. The dependent variable is a dummy that equals one if an individual's usual work activities or duties have changed since last month. *Post COVID* is a dummy that is equal to one for the months of March and April 2020. *Cumulative Cases per*10,000 is a variable equal to the number of cumulative number of confirmed COVID-19 cases per 10,000 inhabitants in the state. All columns include year, month and state fixed effects. Columns 2, 3, 5 and 6 add interview type fixed effects and the following demographic controls: gender, age, marital status and race; and the following state COVID-19 related controls: the number of COVID-19 tests performed per 10,000 inhabitants, implementation of mandatory face mask policies, social distancing measures, day care and school closures, and freezes on eviction and utilities. Columns 3 and 6 add education dummies and four Census region × year fixed effects. The time period is January 2016–June 2020.

Table S8: Work Arrangement Impacts of COVID-19: Exposure, Proximity, Remote and Essential Work

Notes: Data from the Current Population Survey. Robust standard errors are in parentheses, adjusted for clustering by state. The dependent variable is a dummy that equals one if an individual's usual work activities or duties have changed since last month. *Post COVID* is a dummy that is equal to one for the months after March 2020. In columns 1, 2, 3, and 4, *Index* is our exposure to disease index, proximity to coworkers index, remote work index, and essential worker index respectively. All columns include state, month, year, interview type and Census region × year fixed effects and the following demographic controls: gender, age, marital status, education and race; and the following state COVID-19 related controls: the number of COVID-19 tests performed per 10,000 inhabitants, implementation of mandatory face mask policies, social distancing measures, day care and school closures, and freezes on eviction and utilities. The time period is January 2016–June 2020.

Table S9: COVID-19 Lagged (2 weeks) Cases, Deaths and Labor Market Outcomes: State-Level

	Unemployed				In labor force			
	(1)	(2)	(3)	(4)	(5)	(6)	(7)	(8)
	Cases		Deaths		Cases		Deaths	
Cum. COVID-19 cases/deaths per 10,000	0.0000282	-0.000360	0.00316	0.00349	0.000171	0.000208	0.00114	-0.00105
	(0.000235)	(0.000390)	(0.00158)	(0.00278)	(0.0000879)	(0.000210)	(0.00101)	(0.00208)
Case/death rate^2		0.00000216		-0.0000301		-0.000000203		0.000199
		(0.00000139)		(0.000183)		(0.000000852)		(0.000165)
Observations	3158372	3158372	240143	240143	4506713	4506713	346554	346554
	Wages				Hours			
	(1)	(2)	(3)	(4)	(5)	(6)	(7)	(8)
	Cases		Deaths		Cases		Deaths	
Cum. COVID-19 cases/deaths per 10,000	0.000680	-0.00937	-0.0118	-0.110	-0.00104	0.00626	-0.111	-0.0998
	(0.00303)	(0.00782)	(0.0639)	(0.130)	(0.00400)	(0.00633)	(0.0365)	(0.0849)
Case/death rate^2		0.0000593		0.00925		-0.0000411		-0.00101
		(0.0000417)		(0.0122)		(0.0000208)		(0.00604)
Observations	374408	374408	26532	26532	2865884	2865884	205459	205459
Indiv. Chars	Yes	Yes	Yes	Yes	Yes	Yes	Yes	Yes
State FE	Yes	Yes	Yes	Yes	Yes	Yes	Yes	Yes
Region × Year FE	Yes	Yes	Yes	Yes	Yes	Yes	Yes	Yes
Month FE	Yes	Yes	Yes	Yes	Yes	Yes	Yes	Yes
Year FE	Yes	Yes	Yes	Yes	Yes	Yes	Yes	Yes
Interview Type FE	Yes	Yes	Yes	Yes	Yes	Yes	Yes	Yes
State COVID-19 Controls	Yes	Yes	Yes	Yes	Yes	Yes	Yes	Yes

Notes: Data from the Current Population Survey. Robust standard errors are in parentheses, adjusted for clustering by state. Columns 1–2 and 5–6 correspond to cumulative cases per 10,000 while 3–4 and 7–8 correspond to deaths per 10,000. In the top panel, columns 1–4, the dependent variable is a dummy for whether the individual is unemployed. In the top panel, columns 5–8, the dependent variable is a dummy for whether the individual is in the labor force; were at work; held a job but were temporarily absent from work due to factors like vacation or illness; were seeking work; or were temporarily laid off from a job during the reference period. In the bottom panel, columns 1–4, the dependent variable is hours of work for individuals who are employed and either at work or absent from work during the survey week, all jobs. In the bottom panel, column 5–8, the dependent variable is the hourly wages for individuals currently employed as wage/salary workers, paid hourly, and were in outgoing rotation groups. *Cumulative cases/deaths per*10,000 is a variable equal to the number of cumulative number of confirmed COVID-19 cases or deaths per 10,000 inhabitants in the state. All columns include state, month, year, region × year, and interview type fixed effects and the following demographic controls: gender, age, education marital status and race; and the following state COVID-19 related controls: the number of COVID-19 tests performed per 10,000 inhabitants, implementation of mandatory face mask policies, social distancing measures, day care and school closures, and freezes on eviction and utilities. The time period is January 2016–June 2020.

Table S10: COVID-19 Lagged (2 weeks) Cases and Exposure, Proximity, Remote and Essential Work: State-Level

	Unemployment			
	(1) Exposure	(2) Proximity	(3) Remote	(4) Essential
Known cases per 10,000 lagged 2 weeks	0.00076	0.00076	0.00081	0.00075
	(0.00010)	(0.00011)	(0.00011)	(0.00010)
Index	-0.00368	0.00344	-0.00888	0.00011
	(0.00027)	(0.00034)	(0.00034)	(0.00022)
Index × Lagged Cases	0.00000	0.00033	-0.00035	-0.00019
	(0.00003)	(0.00009)	(0.00005)	(0.00004)
Observations	3146035	3146035	3029863	3028575
	Labor Force Participation			
	(1) Exposure	(2) Proximity	(3) Remote	(4) Essential
Known cases per 10,000 lagged 2 weeks	-0.00002	-0.00002	-0.00002	-0.00002
	(0.00001)	(0.00001)	(0.00001)	(0.00001)
Index	0.00015	-0.00105	0.00148	0.00029
	(0.00005)	(0.00006)	(0.00009)	(0.00010)
Index × Lagged Cases	-0.00000	-0.00002	0.00002	0.00001
	(0.00001)	(0.00001)	(0.00001)	(0.00000)
Observations	3179089	3179089	3061498	3060155
Indiv. Chars	Yes	Yes	Yes	Yes
State FE	Yes	Yes	Yes	Yes
Region × Year FE	Yes	Yes	Yes	Yes
Month FE	Yes	Yes	Yes	Yes
Year FE	Yes	Yes	Yes	Yes
Interview Type FE	Yes	Yes	Yes	Yes
State COVID-19 Controls	Yes	Yes	Yes	Yes

Notes: Data from the Current Population Survey. Robust standard errors are in parentheses, adjusted for clustering by state. In the top panel, the dependent variable is a dummy for whether the individual is unemployed. In the bottom panel, the dependent variable is a dummy for whether the individual is in the labor force; were at work; held a job but were temporarily absent from work due to factors like vacation or illness; were seeking work; or were temporarily laid off from a job during the reference period. $Cumulative\ Cases\ per 10,000$ is a variable equal to the number of cumulative number of confirmed COVID-19 cases per 10,000 inhabitants in the state. In columns 1, 3 and 5, *Index* is our exposure to disease index, proximity to coworkers index and remote work index, respectively. All columns include state, month, year, interview type and Census region × year fixed effects and the following demographic controls: gender, age, marital status, education and race; and the following state COVID-19 related controls: the number of COVID-19 tests performed per 10,000 inhabitants, implementation of mandatory face mask policies, social distancing measures, day care and school closures, and freezes on eviction and utilities. The time period is January 2016–June 2020.

Table S11: COVID-19 Lagged (2 weeks) Cases and Exposure, Proximity, Remote and Essential Work: State-Level

	Wages			
	(1)	(2)	(3)	(4)
Known cases per 10,000 lagged 2 weeks	0.00690	0.00642	0.00753	0.00769
	(0.00143)	(0.00121)	(0.00105)	(0.00108)
Index	1.01151	-0.21699	1.35722	0.39310
	(0.03457)	(0.03402)	(0.09416)	(0.02188)
Index × Lagged Cases	-0.00058	0.00184	0.00081	-0.00145
	(0.00154)	(0.00158)	(0.00148)	(0.00257)
Observations	374408	374408	359578	359535
	Hours			
	(1) Exposure	(2) Proximity	(3) Remote	(4) Essential
Known cases per 10,000 lagged 2 weeks	-0.00459	-0.00503	-0.00532	-0.00500
	(0.00154)	(0.00161)	(0.00157)	(0.00169)
Index	-0.30443	-0.98881	0.99716	0.25898
	(0.03503)	(0.05058)	(0.03441)	(0.04510)
Index × Lagged Cases	-0.00071	0.00014	0.00137	-0.00606
	(0.00117)	(0.00133)	(0.00073)	(0.00107)
Observations	2865884	2865884	2759760	2758846
Indiv. Chars	Yes	Yes	Yes	Yes
State FE	Yes	Yes	Yes	Yes
Region × Year FE	Yes	Yes	Yes	Yes
Month FE	Yes	Yes	Yes	Yes
Year FE	Yes	Yes	Yes	Yes
Interview Type FE	Yes	Yes	Yes	Yes
State COVID-19 Controls	Yes	Yes	Yes	Yes

Notes: Data from the Current Population Survey. Robust standard errors are in parentheses, adjusted for clustering by state. In the top panel, the dependent variable is the hourly wages for individuals currently employed as wage/salary workers, paid hourly, and were in outgoing rotation groups. In the bottom panel, the dependent variable is hours of work for individuals who are employed and either at work or absent from work during the survey week, all jobs. *Cumulative Cases per*10,000 is a variable equal to the number of cumulative number of confirmed COVID-19 cases per 10,000 inhabitants in the state. In columns 1, 3 and 5, *Index* is our exposure to disease index, proximity to coworkers index and remote work index, respectively. All columns include state, month, year, interview type and Census region × year fixed effects and the following demographic controls: gender, age, marital status, education and race; and the following state COVID-19 related controls: the number of COVID-19 tests performed per 10,000 inhabitants, implementation of mandatory face mask policies, social distancing measures, day care and school closures, and freezes on eviction and utilities. The time period is January 2016–June 2020.

Table S12: COVID-19 Lagged (2 weeks) Deaths and Exposure, Proximity, Remote and Essential Work: State-Level

	Unemployment			
	(1) Exposure	(2) Proximity	(3) Remote	(4) Essential
Deaths per 10,000 lagged 2 weeks	0.00444	0.00442	0.00484	0.00449
	(0.00070)	(0.00074)	(0.00083)	(0.00079)
Index	-0.00523	0.01542	-0.02210	-0.00855
	(0.00096)	(0.00130)	(0.00122)	(0.00125)
Index × Lagged Deaths	0.00006	0.00286	-0.00307	-0.00138
	(0.00054)	(0.00104)	(0.00038)	(0.00077)
Observations	239427	239427	229697	229594
	Labor Force Participation			
	(1) Exposure	(2) Proximity	(3) Remote	(4) Essential
Deaths per 10,000 lagged 2 weeks	0.00042	0.00043	0.00050	0.00051
	(0.00015)	(0.00014)	(0.00015)	(0.00015)
Index	0.00002	-0.00223	0.00275	0.00041
	(0.00028)	(0.00025)	(0.00031)	(0.00030)
Index × Lagged Deaths	0.00000	-0.00014	0.00011	0.00011
	(0.00008)	(0.00011)	(0.00006)	(0.00007)
Observations	242830	242830	232992	232884
Indiv. Chars	Yes	Yes	Yes	Yes
State FE	Yes	Yes	Yes	Yes
Region × Year FE	Yes	Yes	Yes	Yes
Month FE	Yes	Yes	Yes	Yes
Year FE	Yes	Yes	Yes	Yes
Interview Type FE	Yes	Yes	Yes	Yes
State COVID-19 Controls	Yes	Yes	Yes	Yes

Notes: Data from the Current Population Survey. Robust standard errors are in parentheses, adjusted for clustering by state. In the top panel, the dependent variable is a dummy for whether the individual is unemployed. In the bottom panel, the dependent variable is a dummy for whether the individual is in the labor force; were at work; held a job but were temporarily absent from work due to factors like vacation or illness; were seeking work; or were temporarily laid off from a job during the reference period. *Cumulative deaths per*$10,000$ is a variable equal to the number of cumulative number of confirmed COVID-19 deaths per 10,000 inhabitants in the state. *Index* is our exposure to disease index, proximity to coworkers index, essential worker and remote work index, respectively. All columns include state, month, year, interview type and Census region × year fixed effects and the following demographic controls: gender, age, marital status, education and race; and the following state COVID-19 related controls: the number of COVID-19 tests performed per 10,000 inhabitants, implementation of mandatory face mask policies, social distancing measures, day care and school closures, and freezes on eviction and utilities. The time period is January 2016–June 2020.

Table S13: COVID-19 Lagged (2 weeks) Deaths and Exposure, Proximity, Remote and Essential Work: State-Level

	Wages			
	(1) Exposure	(2) Proximity	(3) Remote	(4) Essential
Deaths per 10,000 lagged 2 weeks	0.06446	0.06379	0.07667	0.06591
	(0.03657)	(0.03691)	(0.03221)	(0.03605)
Index	0.97748	-0.07643	1.28993	0.15480
	(0.07977)	(0.06374)	(0.07894)	(0.09766)
Index × Lagged Deaths	-0.00222	-0.00566	0.02690	0.02900
	(0.02123)	(0.02018)	(0.01882)	(0.05162)
Observations	26532	26532	25492	25490
	Hours			
	(1) Exposure	(2) Proximity	(3) Remote	(4) Essential
Deaths per 10,000 lagged 2 weeks	0.01941	0.01838	0.01942	0.01955
	(0.01864)	(0.01667)	(0.01807)	(0.01971)
Index	-0.46145	-1.06783	1.00059	-0.04281
	(0.06667)	(0.05936)	(0.06472)	(0.05740)
Index × Lagged Deaths	-0.00238	0.00870	0.01916	-0.04247
	(0.01946)	(0.02021)	(0.01128)	(0.01050)
Observations	205459	205459	196838	196771
Indiv. Chars	Yes	Yes	Yes	Yes
State FE	Yes	Yes	Yes	Yes
Region × Year FE	Yes	Yes	Yes	Yes
Month FE	Yes	Yes	Yes	Yes
Year FE	Yes	Yes	Yes	Yes
Interview Type FE	Yes	Yes	Yes	Yes
State COVID-19 Controls	Yes	Yes	Yes	Yes

Notes: Data from the Current Population Survey. Robust standard errors are in parentheses, adjusted for clustering by state. In the top panel, the dependent variable is the hourly wages for individuals currently employed as wage/salary workers, paid hourly, and were in outgoing rotation groups. In the bottom panel, the dependent variable is hours of work for individuals who are employed and either at work or absent from work during the survey week, all jobs. *Cumulative deaths per*10,000 is a variable equal to the number of cumulative number of confirmed COVID-19 deaths per 10,000 inhabitants in the state. *Index* is our exposure to disease index, proximity to coworkers index, essential worker, and remote work index, respectively. All columns include state, month, year, interview type and Census region × year fixed effects and the following demographic controls: gender, age, marital status, education and race; and the following state COVID-19 related controls: the number of COVID-19 tests performed per 10,000 inhabitants, implementation of mandatory face mask policies, social distancing measures, day care and school closures, and freezes on eviction and utilities. The time period is January 2016–June 2020.

Table S14: The Impacts of Lagged (2 weeks) Cases: Exposure, Proximity, Remote and Essential Work

	Unemployed	LFP	Wages	Hours
Known cases per 10,000 lagged 2 weeks	0.0000	0.0000	0.0031	0.0072
	(0.0001)	(0.0000)	(0.0027)	(0.0024)
Exposure	-0.0096	0.0014	1.8023	0.5832
	(0.0005)	(0.0001)	(0.0393)	(0.0311)
Exposure × Lagged Case Rate	-0.0003	0.0000	-0.0001	0.0001
	(0.0001)	(0.0000)	(0.0022)	(0.0011)
Proximity	0.0054	-0.0015	-0.8561	-1.2929
	(0.0007)	(0.0001)	(0.0380)	(0.0588)
Proximity × Lagged Case Rate	0.0005	-0.0000	-0.0015	0.0011
	(0.0002)	(0.0000)	(0.0029)	(0.0019)
Remote Work	-0.0093	0.0012	1.5422	0.6394
	(0.0005)	(0.0001)	(0.0912)	(0.0327)
Remote Work × Lagged Case Rate	-0.0003	0.0000	0.0004	0.0010
	(0.0000)	(0.0000)	(0.0025)	(0.0013)
Essential Worker	-0.0008	0.0005	0.4328	0.4220
	(0.0002)	(0.0001)	(0.0243)	(0.0475)
Essential Worker × Lagged Case Rate	-0.0003	0.0000	-0.0006	-0.0040
	(0.0001)	(0.0000)	(0.0036)	(0.0010)
Observations	3028575	3060155	359535	2758846
Indiv. Chars	Yes	Yes	Yes	Yes
State FE	Yes	Yes	Yes	Yes
Region × Year FE	Yes	Yes	Yes	Yes
Month FE	Yes	Yes	Yes	Yes
Year FE	Yes	Yes	Yes	Yes
Interview Type FE	Yes	Yes	Yes	Yes
State COVID-19 Controls	Yes	Yes	Yes	Yes

Notes: Data from the Current Population Survey. Robust standard errors are in parentheses, adjusted for clustering by state. In column 1 the dependent variable is a dummy for whether the individual is unemployed. In column 2 the dependent variable is a dummy for whether the individual is in the labor force; were at work; held a job but were temporarily absent from work due to factors like vacation or illness; were seeking work; or were temporarily laid off from a job during the reference period. In the third column, the dependent variable is the hourly wages for individuals currently employed as wage/salary workers, paid hourly, and were in outgoing rotation groups. In the fourth column, the dependent variable is hours of work for individuals who are employed and either at work or absent from work during the survey week, all jobs. $Exposure$ measures our exposure to disease index, $Proximity$ is the proximity to coworkers index, $RemoteWork$ is the remote work index, and $EssentialWorker$ is our essential worker index, respectively. $Cases$ is the number of known COVID-19 cases per 10,000 inhabitants. All columns include state, month, year, interview type and Census region × year fixed effects; the following demographic controls: gender, age, marital status, education and race; and the following state COVID-19 related controls: the number of COVID-19 tests performed per 10,000 inhabitants, implementation of mandatory face mask policies, social distancing measures, day care and school closures, and freezes on eviction and utilities. The time period is January 2016–June 2020.

Table S15: The Impacts of Lagged (2 weeks) Deaths: Exposure, Proximity, Remote and Essential Work

	Unemployed	LFP	Wages	Hours
Deaths per 10,000 lagged 2 weeks	0.0037	0.0010	0.0036	-0.1090
	(0.0014)	(0.0004)	(0.0609)	(0.0355)
Exposure	-0.0176	0.0011	1.0458	0.0468
	(0.0017)	(0.0004)	(0.0881)	(0.0700)
Exposure index × Lagged Death Rate	-0.0025	0.0001	0.0029	-0.0063
	(0.0005)	(0.0002)	(0.0276)	(0.0180)
Proximity	0.0206	-0.0023	-0.4139	-1.0251
	(0.0019)	(0.0004)	(0.0705)	(0.0840)
Proximity × Lagged Death Rate	0.0039	-0.0003	-0.0571	0.0242
	(0.0017)	(0.0003)	(0.0315)	(0.0270)
Remote Work	-0.0141	0.0006	0.8264	0.0618
	(0.0013)	(0.0004)	(0.0609)	(0.0540)
Remote Work × Lagged Death Rate	-0.0020	0.0000	-0.0156	0.0091
	(0.0008)	(0.0001)	(0.0309)	(0.0215)
Essential Worker	-0.0147	0.0010	0.3088	0.2709
	(0.0013)	(0.0003)	(0.0684)	(0.0576)
Essential Worker × Lagged Death Rate	-0.0023	0.0002	0.0239	-0.0321
	(0.0007)	(0.0001)	(0.0605)	(0.0130)
Observations	229594	232884	25490	196771
Indiv. Chars	Yes	Yes	Yes	Yes
State FE	Yes	Yes	Yes	Yes
Region × Year FE	Yes	Yes	Yes	Yes
Month FE	Yes	Yes	Yes	Yes
Year FE	Yes	Yes	Yes	Yes
Interview Type FE	Yes	Yes	Yes	Yes
State COVID-19 Controls	Yes	Yes	Yes	Yes

Notes: Data from the Current Population Survey. Robust standard errors are in parentheses, adjusted for clustering by state. In column 1 the dependent variable is a dummy for whether the individual is unemployed. In column 2 the dependent variable is a dummy for whether the individual is in the labor force; were at work; held a job but were temporarily absent from work due to factors like vacation or illness; were seeking work; or were temporarily laid off from a job during the reference period. In the third column, the dependent variable is the hourly wages for individuals currently employed as wage/salary workers, paid hourly, and were in outgoing rotation groups. In the fourth column, the dependent variable is hours of work for individuals who are employed and either at work or absent from work during the survey week, all jobs. *Exposure* measures our exposure to disease index, *Proximity* is the proximity to coworkers index, *RemoteWork* is the remote work index, and *EssentialWorker* is our essential worker index, respectively. *Deaths* is the number of COVID-19 deaths per 10,000 inhabitants. All columns include state, month, year, interview type and Census region × year fixed effects; the following demographic controls: gender, age, marital status, education and race; and the following state COVID-19 related controls: the number of COVID-19 tests performed per 10,000 inhabitants, implementation of mandatory face mask policies, social distancing measures, day care and school closures, and freezes on eviction and utilities. The time period is January 2016–June 2020.

Table S16: The Impacts of Cases: Healthcare and Non-Healthcare Essential Workers

	Essential Worker	Healthcare Essential Worker	Non-Healthcare Essential Worker
Panel A. Unemployed			
Index	-0.0013	-0.0092	-0.0009
	(0.0002)	(0.0010)	(0.0002)
Known cases per 10,000 lagged 2 weeks	0.0000	0.0001	0.0000
	(0.0002)	(0.0002)	(0.0002)
Index × Lagged Case Rate	-0.0002	-0.0006	-0.0002
	(0.0000)	(0.0001)	(0.0000)
Observations	3028575	3154563	3032384
Panel B. Labor Force Participation			
Index	0.0003	0.1573	0.0003
	(0.0001)	(0.0054)	(0.0001)
Known cases per 10,000 lagged 2 weeks	0.0001	0.0002	0.0001
	(0.0000)	(0.0001)	(0.0000)
Index × Lagged Case Rate	0.0000	0.0000	0.0000
	(0.0000)	(0.0001)	(0.0000)
Observations	3060155	4502853	3064015
Panel C. Wages			
Index	0.4893	2.4779	0.3538
	(0.0202)	(0.1340)	(0.0236)
Known cases per 10,000 lagged 2 weeks	0.0022	0.0019	0.0017
	(0.0028)	(0.0029)	(0.0029)
Index × Lagged Case Rate	-0.0017	-0.0110	-0.0014
	(0.0026)	(0.0095)	(0.0028)
Observations	359535	373639	360304
Panel D. Hours			
Index	0.3274	-0.5170	0.3785
	(0.0398)	(0.1123)	(0.0436)
Known cases per 10,000 lagged 2 weeks	-0.0009	-0.0010	-0.0010
	(0.0043)	(0.0040)	(0.0043)
Index × Lagged Case Rate	-0.0052	-0.0023	-0.0053
	(0.0009)	(0.0028)	(0.0009)
Observations	2758846	2862358	2762372
Indiv. Chars	Yes	Yes	Yes
State FE	Yes	Yes	Yes
Interview Type FE	Yes	Yes	Yes
State COVID-19 Controls	Yes	Yes	Yes
Region × Year FE	Yes	Yes	Yes
Month FE	Yes	Yes	Yes
Year FE	Yes	Yes	Yes

Notes: Data from the Current Population Survey. Robust standard errors are in parentheses, adjusted for clustering by state. In the top panel, the dependent variable is a dummy for whether the individual is unemployed. In the second, the dependent variable is a dummy for whether the individual is in the labor force; were at work; held a job but were temporarily absent from work due to factors like vacation or illness; were seeking work; or were temporarily laid off from a job during the reference period. In the third panel, the dependent variable is the hourly wages for individuals currently employed as wage/salary workers, paid hourly, and were in outgoing rotation groups. In the bottom panel, the dependent variable is hours of work for individuals who are employed and either at work or absent from work during the survey week, all jobs. *Index* measures is our essential worker index for the version denoted in the column titles. Column 1 contains estimates for all essential workers, column 2 for essential workers in healthcare, and column 3 for essential workers not in healthcare. *Cases* is the number of known COVID-19 cases per 10,000 inhabitants. All columns include state, month, year, interview type and Census region × year fixed effects; the following demographic controls: gender, age, marital status, education and race; and the following state COVID-19 related controls: the number of COVID-19 tests performed per 10,000 inhabitants, implementation of [illegible]

Table S17: The Impacts of Deaths: Healthcare and Non-Healthcare Essential Workers

Panel A. Unemployed

	Essential Worker	Healthcare Essential Worker	Non-Healthcare Essential Worker
Index	-0.0123	-0.0326	-0.0111
	(0.0013)	(0.0047)	(0.0012)
Deaths per 10,000 lagged 2 weeks	0.0036	0.0034	0.0036
	(0.0016)	(0.0016)	(0.0016)
Index × Lagged Death Rate	-0.0015	-0.0058	-0.0012
	(0.0008)	(0.0021)	(0.0008)
Observations	229594	239933	229804

Panel B. Labor Force Participation

	Essential Worker	Healthcare Essential Worker	Non-Healthcare Essential Worker
Index	0.0008	0.1551	0.0006
	(0.0003)	(0.0062)	(0.0003)
Deaths per 10,000 lagged 2 weeks	0.0010	0.0010	0.0010
	(0.0004)	(0.0010)	(0.0004)
Index × Lagged Death Rate	0.0001	0.0007	0.0001
	(0.0001)	(0.0020)	(0.0001)
Observations	232884	346337	233101

Panel C. Wages

	Essential Worker	Healthcare Essential Worker	Non-Healthcare Essential Worker
Index	0.2918	1.3544	0.1984
	(0.0643)	(0.3431)	(0.0641)
Deaths per 10,000 lagged 2 weeks	0.0016	-0.0148	-0.0008
	(0.0628)	(0.0612)	(0.0638)
Index × Lagged Death Rate	0.0200	0.0379	0.0158
	(0.0467)	(0.1002)	(0.0536)
Observations	25490	26494	25528

Panel D. Hours

	Essential Worker	Healthcare Essential Worker	Non-Healthcare Essential Worker
Index	0.0989	-0.9257	0.1716
	(0.0578)	(0.1688)	(0.0580)
Deaths per 10,000 lagged 2 weeks	-0.1130	-0.1118	-0.1140
	(0.0369)	(0.0361)	(0.0372)
Index × Lagged Death Rate	-0.0403	0.0267	-0.0449
	(0.0112)	(0.0345)	(0.0122)
Observations	196771	205268	196962
Indiv. Chars	Yes	Yes	Yes
State FE	Yes	Yes	Yes
Interview Type FE	Yes	Yes	Yes
State COVID-19 Controls	Yes	Yes	Yes
Region × Year FE	Yes	Yes	Yes
Month FE	Yes	Yes	Yes
Year FE	Yes	Yes	Yes

Notes: Data from the Current Population Survey. Robust standard errors are in parentheses, adjusted for clustering by state. In the top panel, the dependent variable is a dummy for whether the individual is unemployed. In the second, the dependent variable is a dummy for whether the individual is in the labor force; were at work; held a job but were temporarily absent from work due to factors like vacation or illness; were seeking work; or were temporarily laid off from a job during the reference period. In the third panel, the dependent variable is the hourly wages for individuals currently employed as wage/salary workers, paid hourly, and were in outgoing rotation groups. In the bottom panel, the dependent variable is hours of work for individuals who are employed and either at work or absent from work during the survey week, all jobs. *Index* measures is our essential worker index for the version denoted in the column titles. Column 1 contains estimates for all essential workers, column 2 for essential workers in healthcare, and column 3 for essential workers not in healthcare. *Deaths* is the number of COVID-19 deaths per 10,000 inhabitants. All columns include state, month, year, interview type and Census region × year fixed effects; the following demographic controls: gender, age, marital status, education and race; and the following state COVID-19 related controls: the number of COVID-19 tests performed per 10,000 inhabitants, implementation of [illegible]

Table S18: The Impacts of COVID-19: Exposure, Proximity, Remote Work and Essential Workers Exclusive Index Scores

	Unemployed	LFP	Wages	Hours
Post COVID-19	0.0199	-0.0014	-0.0622	-0.5000
	(0.0036)	(0.0009)	(0.1985)	(0.0958)
Exposure	-0.0071	0.0006	1.1631	0.1545
	(0.0005)	(0.0001)	(0.0412)	(0.0406)
Exposure × Post	-0.0183	0.0012	-0.1187	0.0503
	(0.0022)	(0.0004)	(0.1178)	(0.0641)
Essential Worker	-0.0016	0.0005	0.2481	0.0461
	(0.0006)	(0.0001)	(0.0373)	(0.0419)
Essential Worker × Post	-0.0083	0.0015	-0.0356	0.2799
	(0.0021)	(0.0007)	(0.1314)	(0.0988)
Remote Work	-0.0059	0.0002	0.7345	-0.1205
	(0.0004)	(0.0002)	(0.0737)	(0.0327)
Remote Work × Post	-0.0123	0.0028	0.0132	0.5652
	(0.0030)	(0.0007)	(0.1389)	(0.0949)
Proximity	0.0024	-0.0008	-0.4071	-0.9065
	(0.0011)	(0.0002)	(0.0364)	(0.0581)
Proximity × Post	0.0248	-0.0012	-0.0014	0.1192
	(0.0030)	(0.0005)	(0.1000)	(0.0775)
Observations	2220174	2242805	272907	2028013
Indiv. Chars	Yes	Yes	Yes	Yes
State FE	Yes	Yes	Yes	Yes
Interview Type FE	Yes	Yes	Yes	Yes
Region × Year FE	Yes	Yes	Yes	Yes
Month FE	Yes	Yes	Yes	Yes
Year FE	Yes	Yes	Yes	Yes
State COVID-19 Controls	Yes	Yes	Yes	Yes

Notes: Data from the Current Population Survey. Robust standard errors are in parentheses, adjusted for clustering by state. In column 1 the dependent variable is a dummy for whether the individual is unemployed. In column 2 the dependent variable is a dummy for whether the individual is in the labor force; were at work; held a job but were temporarily absent from work due to factors like vacation or illness; were seeking work; or were temporarily laid off from a job during the reference period. In the third column, the dependent variable is the hourly wages for individuals currently employed as wage/salary workers, paid hourly, and were in outgoing rotation groups. In the fourth column, the dependent variable is hours of work for individuals who are employed and either at work or absent from work during the survey week, all jobs. *Exposure* measures our exposure to disease index, *Proximity* is the proximity to coworkers index, $RemoteWork$ is the remote work index, and $EssentialWorker$ is our essential worker index, respectively. $Post\ COVID$ is a dummy that is equal to one for the months after March 2020. All columns include state, month, year, interview type and Census region × year fixed effects; the following demographic controls: gender, age, marital status, education and race; and the following state COVID-19 related controls: the number of COVID-19 tests performed per 10,000 inhabitants, implementation of mandatory face mask policies, social distancing measures, day care and school closures, and freezes on eviction and utilities. The time period is January 2016–June 2020.
